# Ligand‐Defined Interfacial Chemistry Enables Instant and Sequence‐General DNA Chemisorption on Gold Nanoparticles Toward Label‐Free SERS With Single‐Base Resolution

**DOI:** 10.1002/advs.75804

**Published:** 2026-06-09

**Authors:** Guangping Li, Cheng Wang, Xinyue Wu, Zhongxiang Ding, Heng Gao, Zhonggang Liu, Yu Zhang, Jinai Chen, Feng Wang, Honglin Liu

**Affiliations:** ^1^ Joint Research Center for Food Derived Functional Factors and Synthetic Biology of IHM, Anhui Provincial International Science and Technology Cooperation Base for Major Metabolic Diseases and Nutritional Interventions, China Light Industry Key Laboratory of Meat Microbial Control and Utilization, School of Food and Biological Engineering Ministry of Education Hefei University of Technology Hefei P. R. China; ^2^ School of Medicine Anhui University of Science and Technology Huainan P. R. China; ^3^ Institutes of Physical Science and Information Technology Anhui University Hefei P. R. China

**Keywords:** DNA chemisorption, gold nanoparticles, interfacial charge regulation, SERS, single‐base resolution, spherical nucleic acids

## Abstract

A coherent mechanistic framework for DNA adsorption on gold nanoparticles (AuNPs) remains elusive, hindering the rational construction of spherical nucleic acids (SNAs). Here, we show that ligand‐defined interfacial charge regulation, achieved using weakly ionized ascorbic acid (AA) ligands, markedly lowers the kinetic barrier for DNA chemisorption on AuNPs. Under near‐neutral conditions, this interface enables rapid, sequence‐general functionalization of AuNPs with both thiolated and non‐thiolated oligonucleotides through simple vortex mixing, even for DNA containing only a single terminal adenine. The AA‐regulated interface also overcomes the long‐standing incompatibility between ‐butanol dehydration and non‐thiolated DNA, enabling sequence‐general SNA formation under dehydration conditions across multiple nanoparticle systems. Mechanistic studies indicate that AA reduces interfacial electrostatic repulsion while promoting hydrogen‐bond‐assisted surface interactions, and base‐substitution experiments identify the adenine N6‐amino group as a critical site for chemisorption. The resulting ordered DNA corona generates uniform plasmonic nanogaps, enabling reproducible, label‐free SERS with single‐base resolution for nucleotide discrimination and cytosine methylation analysis. This work establishes a general interfacial design strategy for constructing uniform plasmonically active nucleic acid nanostructures under mild conditions.

## Graphical abstract

Ascorbic Acid‐Engineered Interface Enables Universal DNA Functionalization on Gold Nanoparticles: Attenuating electrostatic repulsion and leveraging synergistic hydrogen‐bonding interactions significantly lowers the kinetic barrier for DNA adsorption, enabling rapid and sequence‐general functionalization of non‐thiolated DNA‐requiring only a single terminal adenine—while identifying the adenine N6‐amino group as the critical coordination site.

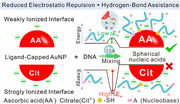

## 1 Introduction

Spherical nucleic acids (SNAs), nanoparticle cores densely functionalized with radially oriented oligonucleotides, have emerged as versatile nanoplatforms for chemical biology, diagnostics, therapeutics, and nanomaterials assembly [1, 2, 3, 4, 5, 6, 7]. By combining programmable base‐pair recognition with the optical/electronic properties of nanostructured cores, SNAs enable molecular sensing, intracellular imaging, and targeted delivery with performance that is often inaccessible to linear nucleic acids [2, 8, 9, 10, 11, 12]. In canonical architectures, gold nanoparticles (AuNPs) are coated with dense shells of single‐stranded DNA, and the performance of SNAs depends critically on the strength and uniformity of the DNA–Au interface [13, 14, 15].

A major challenge is that oligonucleotides are polyanions, whereas colloidal AuNP surfaces are typically stabilized by anionic ligands such as citrate, creating strong electrostatic repulsion that kinetically hinders dense DNA adsorption. Consequently, most high‐density SNAs rely on terminal thiols to form Au─S bonds [16]. However, thiol modification increases synthetic cost and complexity, can be incompatible with certain oligonucleotide libraries, and does not address a broader mechanistic gap [17]. A coherent, predictive framework for how AuNP interfacial chemistry governs sequence compatibility and chemisorption kinetics of nucleic acids remains incomplete, leading to disparate reported driving forces and inconsistent rules for “thiol‐free” attachment.

Considerable efforts have been devoted to overcoming DNA‐AuNP electrostatic barrier by modulating external solution conditions. Salt‐aging increases ionic strength to screen charge repulsion [18, 19], low‐pH transiently protonates nucleobases to reduce repulsion [20, 21], and concentration‐based approaches (freezing, drying, dehydrating) promote adsorption through molecular crowding [17, 22, 23, 24, 25, 26, 27]. Although these methods can produce functional conjugates, they often suffer from multistep processing, long reaction times, variable DNA loading, and pronounced sequence dependence, particularly for non‐thiolated DNA, which typically requires poly(A) tracts or auxiliary motifs for stability. Moreover, butanol dehydration enables ultrafast preparation of thiolated SNAs [25], yet is generally unreliable for non‐thiolated sequences due to colloidal instability [28, 29]. Collectively, these methods primarily “force” adsorption by changing the environment, without directly engineering the intrinsic interfacial properties that define the adsorption energy landscape. This motivates a key question for SNA chemistry: can the AuNP surface itself be rationally redesigned to enable rapid, sequence‐general DNA chemisorption under near‐neutral conditions?

Here, we report that ascorbic acid (AA), used as a weakly ionized capping ligand on gold, provides an interfacial state that dramatically lowers the kinetic barrier for DNA chemisorption and enables spontaneous, rapid, and sequence‐general functionalization at near‐neutral conditions. AA‐capped AuNPs (AA‐AuNPs) support one‐step, vortex‐enabled dense adsorption of both thiolated and non‐thiolated oligonucleotides within seconds. Notably, non‐thiolated DNA can be adsorbed even with only a single terminal adenine, and AA‐AuNPs further render butanol dehydration compatible with non‐thiolated sequences, enabling high‐density SNA formation under dehydration conditions. Mechanistically, AA capping reduces interfacial charge density and promotes hydrogen‐bond‐assisted DNA–surface interactions, thereby attenuating electrostatic repulsion and facilitating DNA adsorption. Base‐substitution experiments further identify the adenine exocyclic N6‐amino group as a critical coordination site, providing molecular evidence for a defined adsorption mode operating without long poly(A) segments. The resulting ordered, upright DNA corona supports uniform plasmonic nanogaps upon assembly, generating highly reproducible, label‐free SERS with single‐base resolution for discriminating single‐nucleotide variants and quantifying cytosine methylation. Together, these results establish ligand‐defined interfacial charge regulation as a practical framework for constructing structurally uniform, plasmonically active nucleic acid nanostructures under biocompatible conditions.

## 2 Results and Discussion

AA‐AuNPs were synthesized via a typical one‐pot reduction method, where ascorbic acid (AA) served as both the reducing and stabilizing agent [30]. Rapid injection (10 mL/s) of AA (100 mm) into preheated HAuCl_4_ (0.25 mm, 80°C) yielded a burgundy colloid within 10 min. The resulting colloid exhibited a surface plasmon resonance (SPR) band centered at 521.0 nm (Figure S1). Transmission Electron Microscopy (TEM) images showed well‐dispersed spherical particles with an average diameter of 23.2 ± 3.8 nm (Figure S2). As a control, citrate‐stabilized AuNPs (Cit‐AuNPs), the most commonly used for SNAs synthesis, were also prepared according to a typical protocol [31].

**FIGURE 1:**
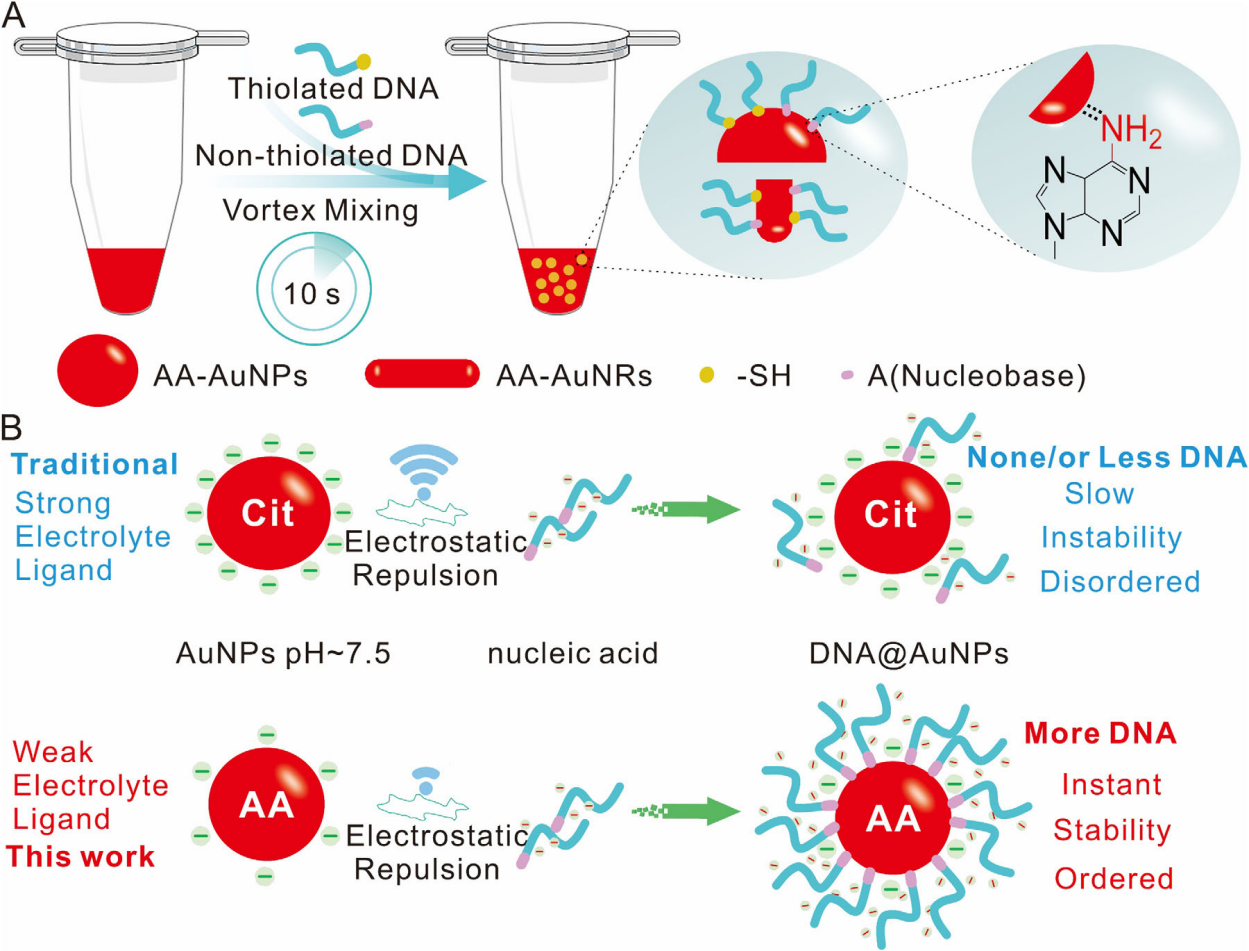
(A) Illustration of universal and instant functionalization of both thiolated and non‐thiolated DNA on AA‐capped surface of both AuNPs and AuNRs, respectively, via one‐step simple vortex mixing, enabled by coordination between the exocyclic N6‐amino group of adenines and the gold surface. (B) Comparison of AA‐ and Cit‐capped AuNPs showing that weakened electrostatic repulsion in AA systems enables instant, dense, and ordered DNA functionalization.

**FIGURE 2:**
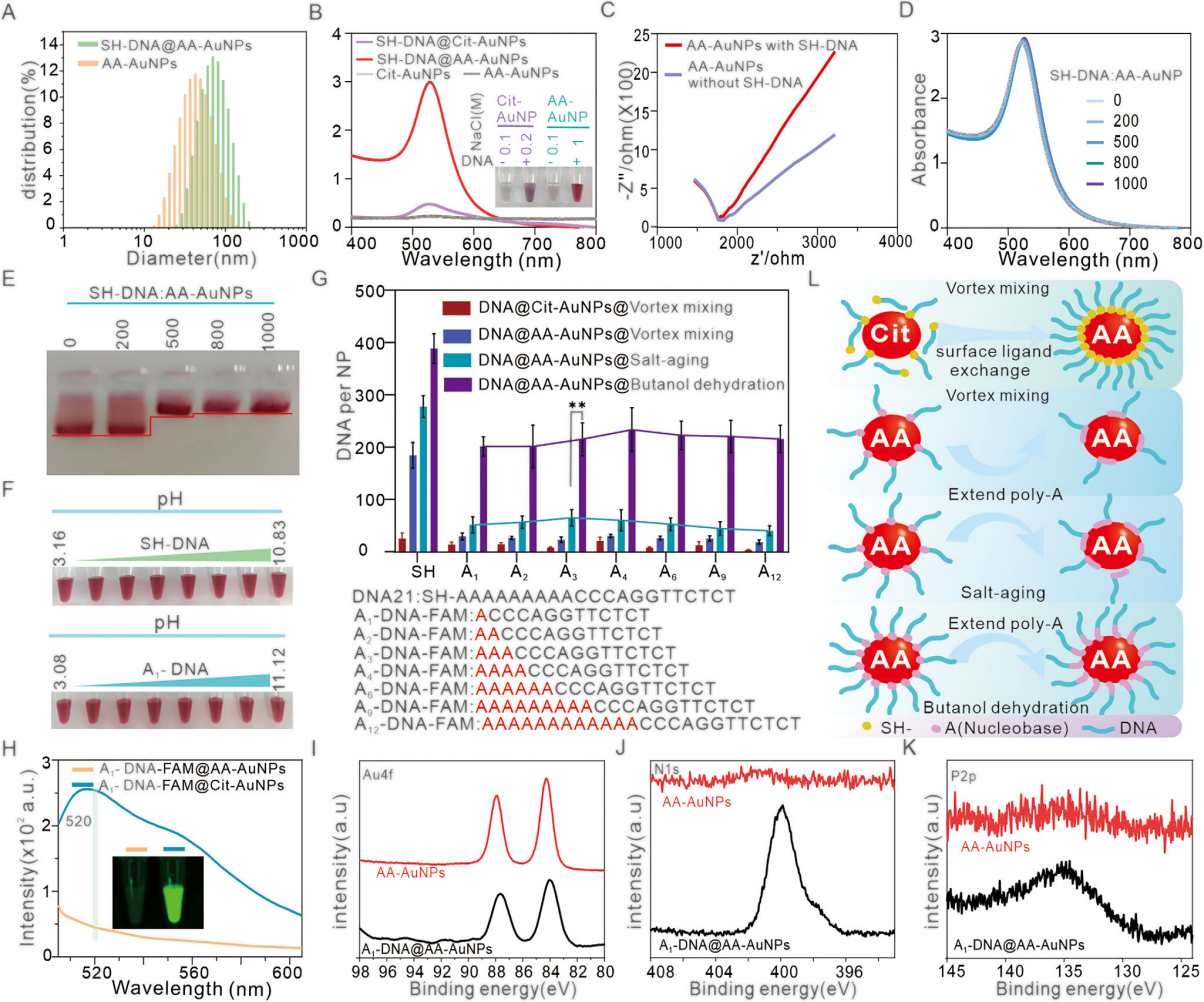
Universal and instant DNA functionalization on AA‐AuNPs. (A) DLS analysis of the hydrodynamic diameters of bare AA‐AuNPs and SH‐DNA@AA‐AuNPs. (B) UV–vis absorbance spectra and optical images of bare Cit‐AuNPs, AA‐AuNPs, SH‐DNA@Cit‐AuNPs, and SH‐DNA@AA‐AuNPs, synthesized by 10 s vortex mixing, in the presence of NaCl at varied concentrations. (C) Nyquist plots showing the electrochemical response of surfaces before and after HS‐DNA chemisorption, using 5 mm [Fe(CN)_6_]^3−/4−^ as redox mediator. (D, E) UV–vis absorbance spectra and agarose gel electrophoresis of SH‐DNA@AA‐AuNPs synthesized at varied SH‐DNA/AA‐AuNP ratios. (F) Stability of thiolated (top) and non‐thiolated (bottom) DNA@AA‐AuNPs across different pH conditions. (G) The amounts of varied DNA strands on different ligand‐capped AuNPs, synthesized by 10 s vortex mixing, butanol dehydration, and salt‐aging at a fixed DNA/AuNP molar ratio of 500:1. Error bars represent the mean ± SD for three independent replicates. ^**^ *p* < 0.01. (H) Fluorescence intensity of fluorophore‐labeled DNA with a single terminal adenine base (A_1_‐DNA‐FAM) in the presence of AA‐AuNPs and Cit‐AuNPs, respectively, after 10 s vortex mixing. The inset shows the fluorescence image under UV illumination. (I–K) XPS spectra illustrated the Au4f, N1s, and P2p elemental analysis of bare AA‐AuNPs and A_1_‐DNA@AA‐AuNPs, respectively. (L) Illustration on the contrasting outcomes of DNA functionalization on AuNPs using vortex mixing, butanol dehydration, and salt‐aging methods with DNA strands bearing short or long poly‐A blocks.

Interestingly, 10 s of simple vortex mixing of 1 nm AA‐AuNPs sols and 500 nm SH‐DNA solution could synthesize SH‐DNA functionalized AA‐AuNPs (labeled as SH‐DNA@AA‐AuNPs) (Figure 1A). The SH‐DNA@AA‐AuNPs yielded an immediate 4 nm redshift in the UV–vis absorbance spectrum (Figure S3), had increased hydrodynamic diameters in dynamic light scattering (DLS), and more negative zeta potentials (Figure 2A, Figure S4A) [5]. Notably, the SH‐DNA@AA‐AuNPs remained stable and well dispersed in 1 m NaCl. In contrast, similar processing could not generate stable SH‐DNA functionalized Cit‐AuNPs, which easily aggregated at 0.2 m NaCl (Figure 2B). It further suggests the spontaneous formation of a stable SH‐DNA shell on AA‐AuNPs.

The difference in interfacial properties was quantified by zeta potential measurements, which yielded values of −39.3 ± 7.0 mV for Cit‐AuNPs and −21.0 ± 5.5 mV for AA‐AuNPs (Figure S5). This marked difference is attributed to the higher charge density imparted by the fully deprotonated citrate (Cit^3^^−^) at pH near‐neutral conditions compared to the singly ionized ascorbate (AA^−^). The significantly lower surface potential of AA‐AuNPs indicates a reduced interfacial charge density, which weakens the electrostatic repulsion between the DNA backbone and the nanoparticle surface, facilitating adsorption. This conclusion was further corroborated by electrochemical impedance spectroscopy (EIS), where a significant increase in charge‐transfer resistance was observed only for AA‐AuNPs upon SH‐DNA addition, while Cit‐AuNPs showed no significant change (Figure 2C, Figure S4B). These results collectively indicate that SH‐DNA chemisorbs preferentially on AA‐AuNPs [32].

The formation of a stable DNA shell on AA‐AuNPs required a minimum DNA concentration; no stable functionalization was detected when the SH‐DNA/AA‐AuNP molar ratio was below 500:1 (Figure 2D,E), consistent with the concentration‐dependent behavior reported for SNA formation [22]. The DNA loading density increased with increasing SH‐DNA concentration and reached saturation at a ratio of 800:1. The resulting SH‐DNA@AA‐AuNPs displayed excellent colloidal stability across a wide pH range (3.1–10.8, Figure 2F).

Quantitative fluorescence measurements further revealed a substantial difference in DNA loading capacity. At an SH‐DNA/AuNP ratio of 500:1, approximately 201 ± 16 DNA strands were immobilized on each AA‐AuNP, compared to only 25 ± 9 strands on Cit‐AuNPs (Figure 2G). These results demonstrate that brief vortex mixing alone enables rapid and dense functionalization of SH‐DNA on AA‐AuNPs, without reliance on salt‐aging, pH modulation, or dehydration‐driven strategies.

Next, non‐thiolated DNA was examined for spontaneous functionalization on the AA‐modified gold surface. Fluorescently labeled DNA with a single terminal adenine base (A_1_‐DNA‐FAM) was directly mixed with AA‐AuNPs and Cit‐AuNPs, respectively. A pronounced fluorescence quenching was immediately observed in the presence of AA‐AuNPs, whereas strong emission persisted in the presence of Cit‐AuNPs (Figure 2H and Movie S1). This contrast indicates that non‐thiolated DNA strands can also spontaneously functionalize on AA‐AuNPs [33, 34, 35]. Quantitative analysis further revealed that, even for poly‐A‐containing DNA (A_12_‐DNA‐FAM), AA‐AuNPs exhibited substantially higher DNA functionalization (18 ± 4 strands per particle) than Cit‐AuNPs (3 ± 1 strands per particle). Remarkably, even DNA sequences with only a single terminal adenine base could undergo spontaneous functionalization on the AA‐AuNPs (Figure 2G). Elemental analysis by X‐ray photoelectron spectroscopy (XPS) provided further support: while the Au4f and C1s signals remained essentially unchanged, distinct N1s and P2p peaks were observed for A_1_‐DNA‐functionalized AA‐AuNPs (Figure 2I–K and Figure S6), confirming the presence of nucleic acids on the AA‐AuNPs surface. The results demonstrate the formation of A_1_‐DNA‐functionalized AA‐AuNPs. The spontaneous functionalization of non‐thiolated DNA on the AA‐AuNPs surface might be attributed to the distinct interfacial interaction, yet the generated colloidal stability might be limited due to the achievable molecular density.

As mentioned above, butanol dehydration enabled flash functionalization of SH‐DNA on Cit‐AuNPs [25], but it is not suitable for non‐thiolated DNA sequences. We speculate that the interfacial environment is crucial in non‐thiolated DNA functionalization on a gold surface. Surprisingly, upon replacing Cit‐AuNPs with AA‐AuNPs, non‐thiolated DNA underwent flash functionalization on AA‐AuNPs using the same butanol dehydration protocol (Figure S7), and this kind of SNAs was very stable and had a very high DNA density that will be discussed later. Specifically, a 13‐mer sequence with only one single A base at the 5' end (A_1_‐DNA) could be well functionalized on AA‐AuNPs via butanol dehydration, which had exceptional colloidal stability in 1.0 m NaCl and across a broad pH range from 3.08 to 11.12(Figure 2F), and even in 80% serum (Figure S8A). A redshift of 3.5 nm in the UV–vis absorbance spectrum relative to bare AA‐AuNPs was observed (Figure S7), consistent with changes in the local dielectric environment upon DNA adsorption. Gel electrophoresis showed a markedly its slower migration than bare AA‐AuNPs, zeta potentials evidenced more negative surface charge that was consistent with DNA shell‐coated AuNPs, and DLS revealed well‐dispersed colloids with a PDI = 0.17 ± 0.02 (Figure S8) [36]. Fluorescence quantification confirmed the successful synthesis of high‐density SNAs with a very high loading of 195 ± 20 A_1_‐DNA‐FAM strands on each AA‐AuNPs particle (Figure 2G).

The effect of terminal poly‐A length on DNA loading onto AA‐AuNPs was examined. Fluorescence calibration curves were first established to ensure the quantitative reliability of the measurements for seven non‐thiolated A_n_‐DNA‐FAM sequences, namely A_1_‐, A_2_‐, A_3_‐, A_4_‐, A_6_‐, A_9_‐, and A_12_‐DNA‐FAM (Figure S9). As plotted in Figure 2G, butanol dehydration enabled the attachment of more than 200 DNA strands per AA‐AuNP for all seven sequences, comparable to the loading levels achieved with SH‐DNA [17]. Previous studies have shown that a poly‐A terminus containing at least two adenine bases is required for non‐thiolated DNA functionalization on Cit‐AuNPs via salt‐aging [17]. But here the salt‐aging could make all of our seven sequences functionalized on AA‐AuNPs with an average of 40–65 DNA molecules per particle, even for the A_1_‐DNA. Interestingly, through the salt‐aging synthesis, DNA loading amounts first increased when increasing the poly‐A length from A_1_ to A_3_, but then gradually decreased from A_3_ to A_12_ with an estimated decrease of 4.3% per A base; In contrast, through the dehydration synthesis, the DNA loading amounts increased when increasing the poly‐A length from A_1_ to A_4_, but then gradually decreased from A_4_ to A_12_ with an estimated decrease of 0.9% per A base (Figure 2G). These results indicate that DNA loading achieved via butanol dehydration is largely insensitive to poly‐A length, exhibiting only minor variation across the examined sequences. This behavior differs markedly from that observed for salt‐aging, suggesting distinct underlying mechanisms. In the salt‐aging process, the DNA loading density remains well below the maximum achievable surface coverage, allowing multiple nucleobases along the poly‐A segment to interact with the gold surface. Although longer poly‐A blocks enhance binding affinity, the associated steric hindrance of the DNA strands can limit the grafting density, reflecting a balance between adsorption affinity and steric effects [19, 37]. In contrast, the dehydration process makes a very high density of DNA loading, and DNA may adsorb mainly through the terminal 5′‐adenine, enabling a lower fluctuation of DNA loading density across varied poly‐A lengths (Figure 2L).

Here, 5 mm NaCl was necessary for the formation of stable SNAs via butanol dehydration. For example, when loading an 11‐mer sequence, A_1_T_10_, with a much lower affinity on AA‐AuNPs, the absence of NaCl led to rapid nanoparticle aggregation, manifested by a purple coloration, whereas the presence of NaCl yielded stable red colloids (Figure S10). This observation indicates that partial screening of electrostatic repulsion between non‐thiolated DNA and AA‐AuNPs facilitates DNA chemisorption. Notably, Cit‐AuNPs and AA‐AuNPs exhibited distinct colloidal behaviors during non‐thiolated DNA functionalization in butanol dehydration. The absence of DNA made rapid aggregation of both bare AA‐AuNPs and Cit‐AuNPs, i.e., butanol disrupts the electrostatic double layer. But the presence of A_1_‐DNA successfully synthesized A_1_‐DNA@AA‐AuNPs, while failing to yield A_1_‐DNA@Cit‐AuNPs (Figure S11 and Movie S2). Even with poly‐A terminal of 12 adenines (A_12_‐DNA), functionalization of Cit‐AuNPs remained unsuccessful (Figure S12). Another notable feature of AA‐AuNPs (23 nm in diameter) is the substantially lower DNA requirement for non‐thiolated DNA loading. Stable SNAs could be obtained at an A_1_‐DNA/AA‐AuNP molar ratio as low as 100:1, even for A_1_‐DNA containing only a single terminal adenine (Figure S13). In comparison, SH‐DNA functionalization of 13 nm Cit‐AuNPs via butanol dehydration typically requires DNA/Cit‐AuNP ratios exceeding 300:1 [25]. Larger AA‐AuNPs usually require much higher DNA/AA‐AuNP molar ratios to achieve stable functionalization (Figure S14), consistent with previous reports [17].

Collectively, these results highlight the fundamentally different interfacial behaviors of AA‐AuNPs and Cit‐AuNPs. AA‐AuNPs enable markedly higher DNA loading, densely packed DNA layers under the butanol dehydration with minimal poly‐A dependence, and require much lower DNA/AuNP ratios to achieve stable functionalization. In contrast, Cit‐AuNPs show strong poly‐A‐dependent loading and often fail to functionalize non‐thiolated DNA. These distinctions indicate that weakly ionized AA ligands create a more permissive, less sequence‐selective surface than citrate, thereby enabling generalizable construction of non‐thiolated SNAs.

To evaluate the universality of AA‐AuNPs for DNA functionalization via butanol dehydration, three representative DNA strands were selected (Table S1), including A_9_‐DNA that cannot be functionalized by the freezing method; A_10_‐DNA1 that enables to functionalize Cit‐AuNPs by the freezing method; and A_10_‐DNA2 that is a 59 nt long sequence and might affect the SNAs stability (Figure S15) [24, 26, 38]. Expectedly, three sequences were successfully and stably functionalized via the use of AA‐AuNPs under butanol dehydration, as evidenced by SPR redshifts relative to bare AA‐AuNPs in the UV–vis absorbance and slower migration in agarose gel electrophoresis (Figure S16). These results highlight that the AA‐mediated interface enables universal and sequence‐independent DNA functionalization, overcoming the pronounced sequence dependence observed in other approaches. Mechanistically, the densely charged citrate layer on Cit‐AuNPs strongly repels DNA, often leading to aggregation, whereas the more weakly ionized AA ligands substantially alleviate this electrostatic barrier. Upon mixing, non‐thiolated DNA can spontaneously adsorb onto AA‐AuNPs, forming an initial protective shell. Subsequent butanol dehydration partially disrupts the interfacial double layer, further reducing electrostatic repulsion both among DNA strands and between DNA and the nanoparticle surface. This process promotes the formation of a compact DNA layer, thereby enhancing colloidal stability and effectively suppressing aggregation.

**FIGURE 3:**
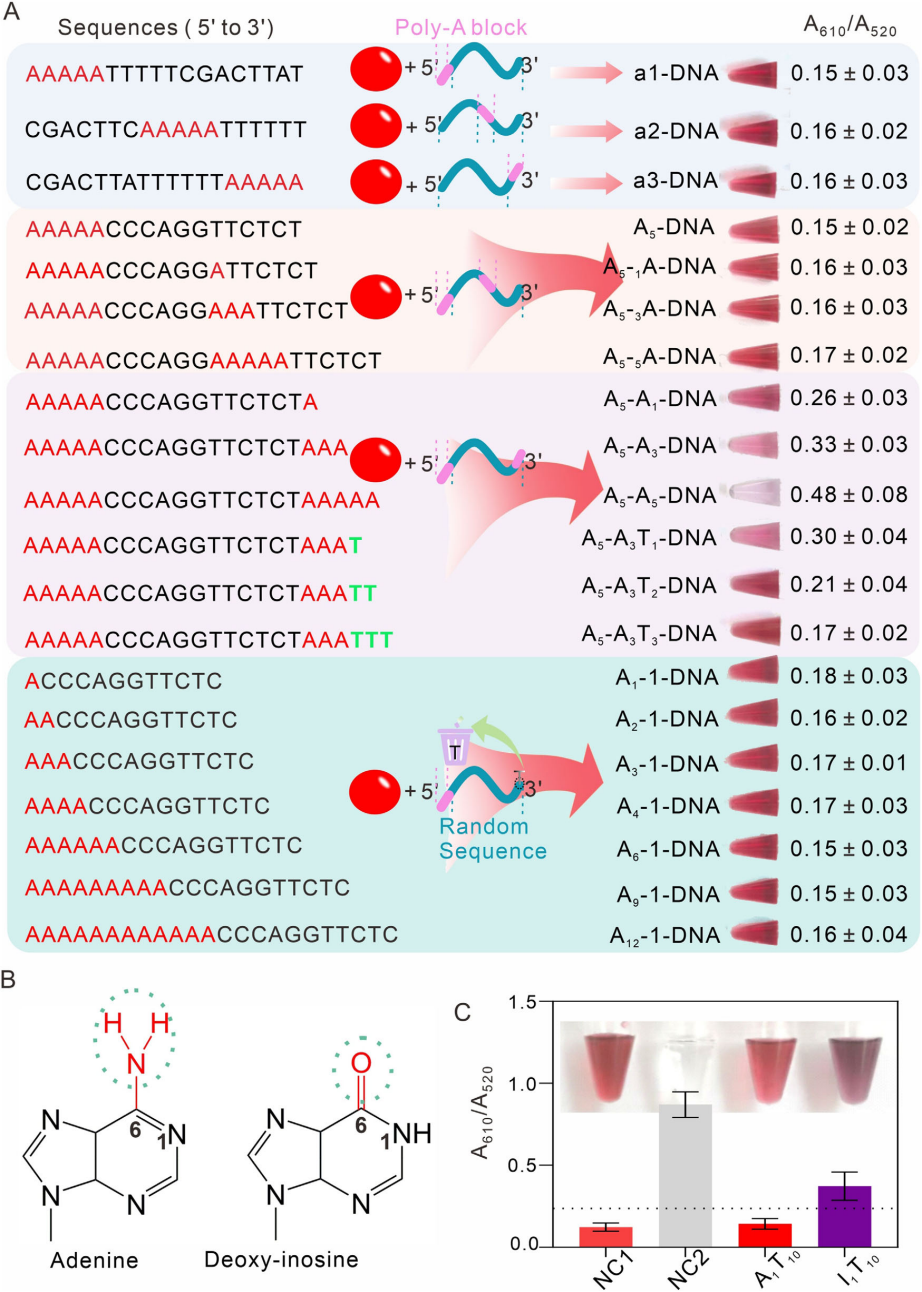
(A) Illustration of sequence‐dependent DNA functionalization on AA‐AuNPs via butanol dehydration and photographs of the resulting colloids. A_610_/A_520_ ratios of AA‐AuNPs functionalized with DNA containing poly‐A segments at different positions. (B) Schematic structures of adenine and deoxy‐inosine. (C) The UV–vis A_610_/A_520_ ratio of X_1_T_10_. The inset shows the colloidal state of the SNAs prepared using X_1_T_10_ (X = A, I) NC1: negative control without the butanol dehydration and any DNA; NC2: negative control with the butanol dehydration but without DNA. Error bars represent the mean ± SD for three independent replicates.

The effects of poly‐A positioning and sequence composition were systematically investigated. In previous experiments, DNA strands bearing a poly‐A block at the 5′ end consistently yielded stable SNAs. Notably, relocating the poly‐A block to the 3′ terminus or to an internal position also resulted in stable SNA formation, as summarized in the blue region of Figure 3A. We further designed DNA sequences containing two poly‐A blocks positioned both in the middle and at the 5′ end. The results also evidenced the formation of stable and dense DNA loading on AA‐AuNPs, as illustrated in the orange region of Figure 3A and Figure S17. In contrast, as illustrated in the purple region of Figure 3A, positioning two poly‐A blocks at both terminals clearly led to nanoparticle aggregation, especially when the number of A bases at both ends tends to be equal, indicating interparticle cross‐linking. But interestingly, the introduction of a single T base at one end could effectively reverse this cross‐linking, and the more T bases were added, the more pronounced the inhibitory effect became. The addition of three T bases is sufficient to completely eliminate this cross‐linking effect, achieving stability comparable to that of A_5_‐DNA that only contains A_5_ at the 5' end, according to the A_610_/A_520_ ratio values. The previous report claimed the outward terminal T base was crucial for synthesizing SNAs through poly‐A chemisorption in microwave heating synthesis [23]. Here, as illustrated in the green region of Figure 3A, all DNA strands do not contain the outward terminal T bases; nevertheless, stable DNA functionalization could also be achieved, further supporting the sequence‐general of the strategy. Collectively, these findings establish that our strategy achieves universal DNA functionalization, with performance unaffected by either the length or the placement of the poly‐A.

To further clarify which base of non‐thiolated DNA interacts with the AA‐AuNPs, four 11‐base sequences, i.e., A_1_A_10_, A_1_C_10_, A_1_G_10,_ and A_1_T_10_, were examined to reveal base‐dependent functionalization on AA‐AuNPs through the butanol dehydration. Results evidenced that the A_1_T_10_ could generate stable SNAs with the AA‐AuNPs, while the others could not generate stable colloids and their stability followed the trend A > C > G > T (Figure S18), supporting a dominant role of the A base in noncovalent DNA‐AA‐AuNPs interactions [17, 39]. Of course, it should be mentioned here that Cit‐AuNPs could not form SNAs with any of the above four sequences. Previous studies have suggested strong A‐base‐gold interactions, while the specific binding atom remains controversial [40, 41, 42, 43]. To further interrogate the functional group responsible for this interaction, the N6 exocyclic amino group of adenine was replaced by a carbonyl through substitution with deoxyinosine, yielding the terminally modified sequence I_1_T_10_ (Figure 3B). But the same butanol dehydration procedure made the color of AA‐AuNPs sols change from red to purple, indicating colloid aggregation (Figure 3C). These results support a critical role of the N6 exocyclic amino group of adenine in mediating coordination with the gold surface.

**FIGURE 4:**
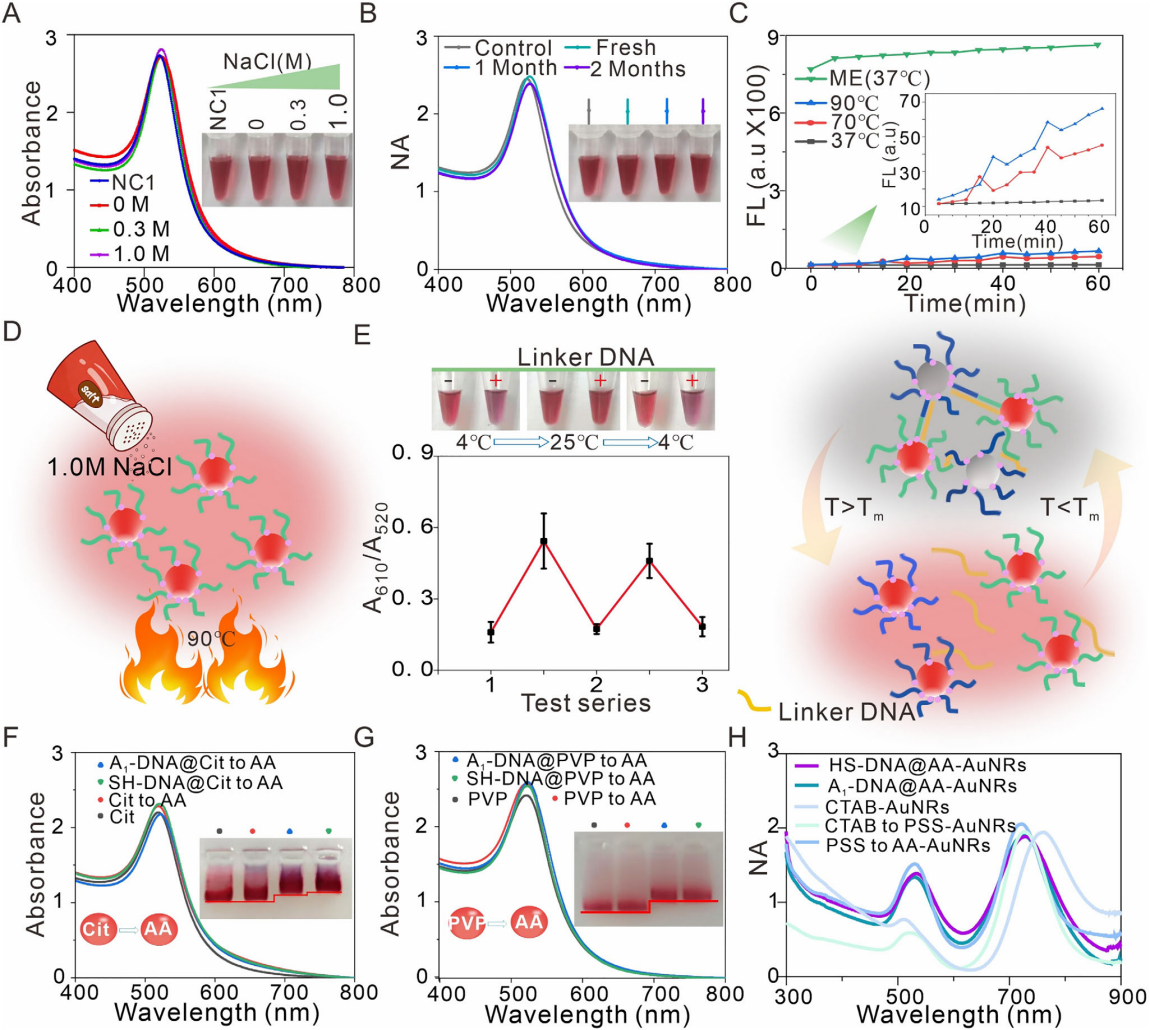
Stability of SNAs. (A) UV–vis absorption spectra of SNAs prepared via the butanol dehydration under high‐salt conditions. Insets show photographs of the corresponding colloidal states. (B) UV–vis monitoring of SNAs stability in 0.3 m NaCl over two months. (C) Fluorescence monitoring of FAM−labeled DNA desorption from AuNPs surfaces at varying temperatures. (D) Schematic illustration showing that SNAs exhibit excellent stability under 1.0 m NaCl conditions or at 90°C. (E) Solution‐based SNAs sensor prepared via the B: photographs under temperature cycling between 4°C and 25°C (top), corresponding UV–vis absorbance ratios (A_610_/A_520_, left), and schematic of the sensing mechanism. Error bars represent the mean ± SD for three independent replicates. UV–vis absorption spectra of DNA‐functionalized gold nanoparticles following ligand exchange from Cit/PVP to AA (F, G). The insets show photographs of the corresponding agarose gel electrophoresis for DNA‐functionalized gold nanoparticles after Cit/PVP‐to‐AA ligand exchange. (H) UV–vis absorption spectra of bare AA‐capped AuNRs and DNA‐functionalized AA‐AuNRs. NA represents normalized absorbance.

As mentioned previously, A_1_‐DNA@AA‐AuNPs exhibited exceptional colloidal stability in 1.0 m NaCl (Figure 4A). Notably, tolerance to 1.0 m NaCl is widely recognized as a hallmark of SNAs with SH‐DNA [17]; here, comparable stability was achieved using the A_1_‐DNA that contains only a single terminal A base. For long‐term stability, the UV–vis absorbance spectra remained unchanged after two months in 0.3 m NaCl (Figure 4B). The exceptional stability under high‐salt and long‐term conditions should originate from a high‐density DNA shell. To exclude the possibility of multilayer DNA adsorption mediated by interstrand hydrogen bonding, thermal stability and competitive desorption assays were conducted. First, FAM‐labeled DNA‐functionalized AA‐AuNPs exhibited no appreciable change in fluorescence intensity over a broad temperature range. Second, the addition of a competitive displacement reagent (mercaptoethanol, ME) induced a sharp increase in fluorescence (Figure 4C), indicative of competitive desorption of DNA strands from the AA‐AuNP surface [23, 44]. These observations rule out nonspecific adsorption and support the high thermal stability of A_1_‐DNA@AA‐AuNPs. Taken together, these results demonstrate the exceptional stability of A_1_‐DNA@AA‐AuNPs under both high‐salt and high‐temperature conditions, a hallmark feature of SNAs, as schematically illustrated in Figure 4D. To verify the hybridization capability of DNA strands on the SNA surface, two batches of non‐thiolated DNA‐functionalized AA‐AuNPs, each carrying a distinct sequence complementary to part of a bifunctional linker, were mixed. Upon addition of the linker, sequence‐specific hybridization induced nanoparticle aggregation, which was fully reversible upon heating. No color change was observed in control samples lacking the linker, confirming that the assembly was entirely driven by DNA base pairing (Figure 4E).

As discussed above, the Cit‐AuNPs could not support spontaneous DNA functionalization, so we speculated that the ligand exchange strategy may extend our SNA preparation method to a broader range of nanoparticle surfaces. Expectedly, the ligand exchange of Cit to AA enabled efficient DNA functionalization (Figure 4F). Importantly, this AA‐directed ligand exchange strategy was successfully extended to other colloidal systems, e.g., PVP‐stabilized AuNPs (Figure 4G) and cetyltrimethylam‐monium bromide (CTAB)‐stabilized Au nanorods (Figure 4H), both of which could be functionalized with non‐thiolated DNA after AA replacement. The TEM images showed unchanged particle morphology and improved dispersion (Figure S19), indicating the successful functionalization of DNA, which is consistent with literature reports.

**FIGURE 5:**
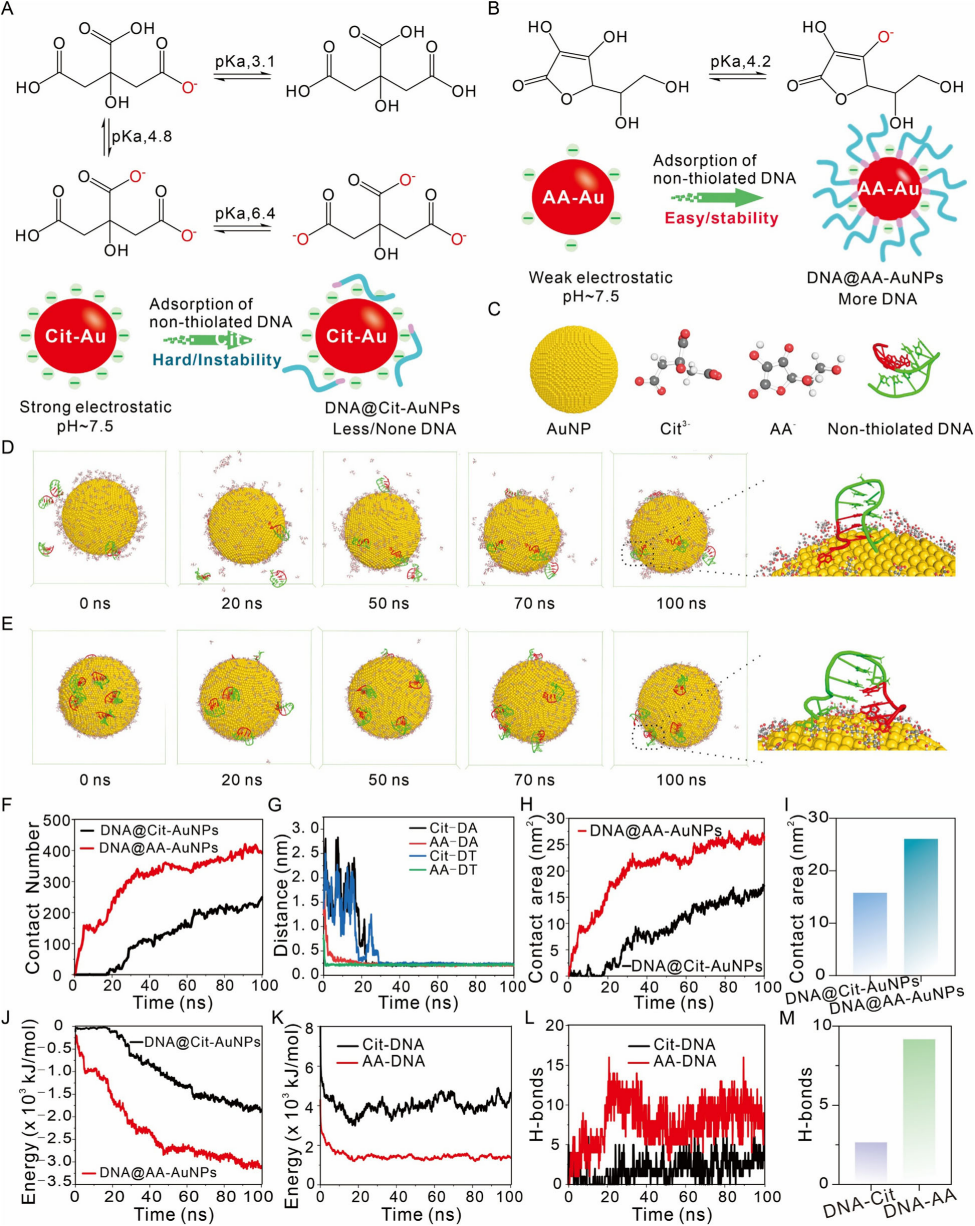
(A) and (B)The ionization of Cit/AA and the schematic illustration of DNA (A_4_T_7_) functionalization Cit­AuNPs and AA­AuNPs. (C) The molecules employed in the molecular dynamics (MD) simulations. (D, E) display representative snapshots of DNA molecules on the surfaces of Cit‐AuNPs and AA‐AuNPs at distinct simulation time points. Water molecules, nonpolar hydrogen atoms, and parts of the DNA structures were omitted for clarity. (F) The number of DNA molecules in contact with the Cit‐AuNPs and AA‐AuNPs surface during the MD simulation. (G) Time‐dependent centroid distance between DNA and Cit/AA‐AuNPs. (H) Time‐dependent contact area between DNA and Cit/AA‐AuNPs. (I) Average contact area over the final 30 ns. (J) Time‐dependent DNA‐AuNPs interaction energy. (K) Time‐dependent electrostatic repulsion between DNA and the two ligand types. (L) Time‐dependent number of hydrogen bonds between DNA and ligands. (M) Average number of hydrogen bonds over the final 30 ns.

To probe the role of ligand ionization at the AuNP interface, all‐atom molecular dynamics simulations were conducted for citrate‐ and ascorbic acid‐capped AuNPs at near‐neutral conditions. To balance the experimental conditions, 10 nm spherical AuNPs were modeled with Cit or AA ligands that ionized as Cit^3−^ and AA^−^, respectively, at a surface density of 1.3 ligands/nm^2^, approximating realistic AuNPs configurations (Figure S20A). Results revealed that the total binding energy of between AA and AuNPs (−75 000 kJ/mol) was approximately 2.3 times larger in magnitude than that of Cit‐AuNPs (−30 000 KJ/mol) (Figure S20B,C). At near‐neutral conditions, citrate is fully deprotonated at its three carboxylate groups (pK_a_ ≈ 3.1, 4.8, 6.4), whereas ascorbate carries a single negative charge originating from its acidic hydroxyl group (pK_a__1_ ≈ 4.2), leading to markedly different surface charge densities (Figure 5A–C). These differences are reflected in the distinct interfacial environments of the two ligand‐stabilized AuNPs.

One hundred DNA strands (A_4_T_7_) were initially placed at a distance of ∼1.0 nm from the AuNP surface, and their trajectories were followed for 100 ns (Figure S21). In the Cit‐AuNPs system, A_4_T_7_ remained largely separated from the gold surface during the initial 20 ns and approached gradually thereafter, with only weak surface association observed (Figure 5D and Movie S4). In contrast, A_4_T_7_ rapidly approached AA‐AuNPs and became stably functionalized (Figure 5E and Movie S3). Quantitative analyses of DNA‐AuNP contacts, center‐of‐mass distances, and contact areas consistently show faster adsorption kinetics and more extensive surface association on AA‐AuNPs (Figure 5F–I). To further assess the interaction characteristics, the DNA‐AuNP interaction was decomposed into total binding energy and electrostatic potential energy components. In the Cit‐AuNPs system, DNA experiences pronounced electrostatic repulsion accompanied by relatively weak binding interactions, whereas in the AA‐AuNPs system, electrostatic repulsion is substantially reduced and binding interactions are strengthened. Notably, the DNA binding energy on AA‐AuNPs remains negative throughout the simulation, consistent with spontaneous DNA functionalization (Figure 5J,K). Hydrogen‐bonding interactions contribute to DNA adsorption on AA‐AuNPs. The hydroxyl groups of AA^−^ ligands can serve as hydrogen‐bond donors, forming directional interactions with DNA phosphate groups and nucleobases. These interactions are associated with reduced DNA mobility near the AuNP surface and facilitate surface association. In contrast, Cit^3^^−^ ligands are less capable of forming sufficient hydrogen bonds due to their high charge density and spatial arrangement (Figure 5L,M), resulting in looser and less favorable DNA binding.

Overall, the surface ligand has a decisive role in governing DNA functionalization on AuNPs. The simulations validated that strongly ionized ligands (Cit^3−^) create a highly charged interface that repels DNA, whereas weakly ionized ligands (AA^−^) form a less charged surface that favors and facilitates SNAs formation. Replacing Cit with AA ligands created a weakly ionized interface that reduced electrostatic repulsion and promoted DNA functionalization. In addition, the hydroxyl groups of AA— may contribute through hydrogen‐bonding interactions with DNA phosphates or nucleobases, while simultaneously modifying the hydration structure at the gold–water interface. Such cooperative interfacial effects may lower the kinetic barrier for DNA anchoring, enabling spontaneous functionalization even for non‐thiolated strands containing only a single terminal adenine (Figure 1B) [19, 37, 45].

**FIGURE 6:**
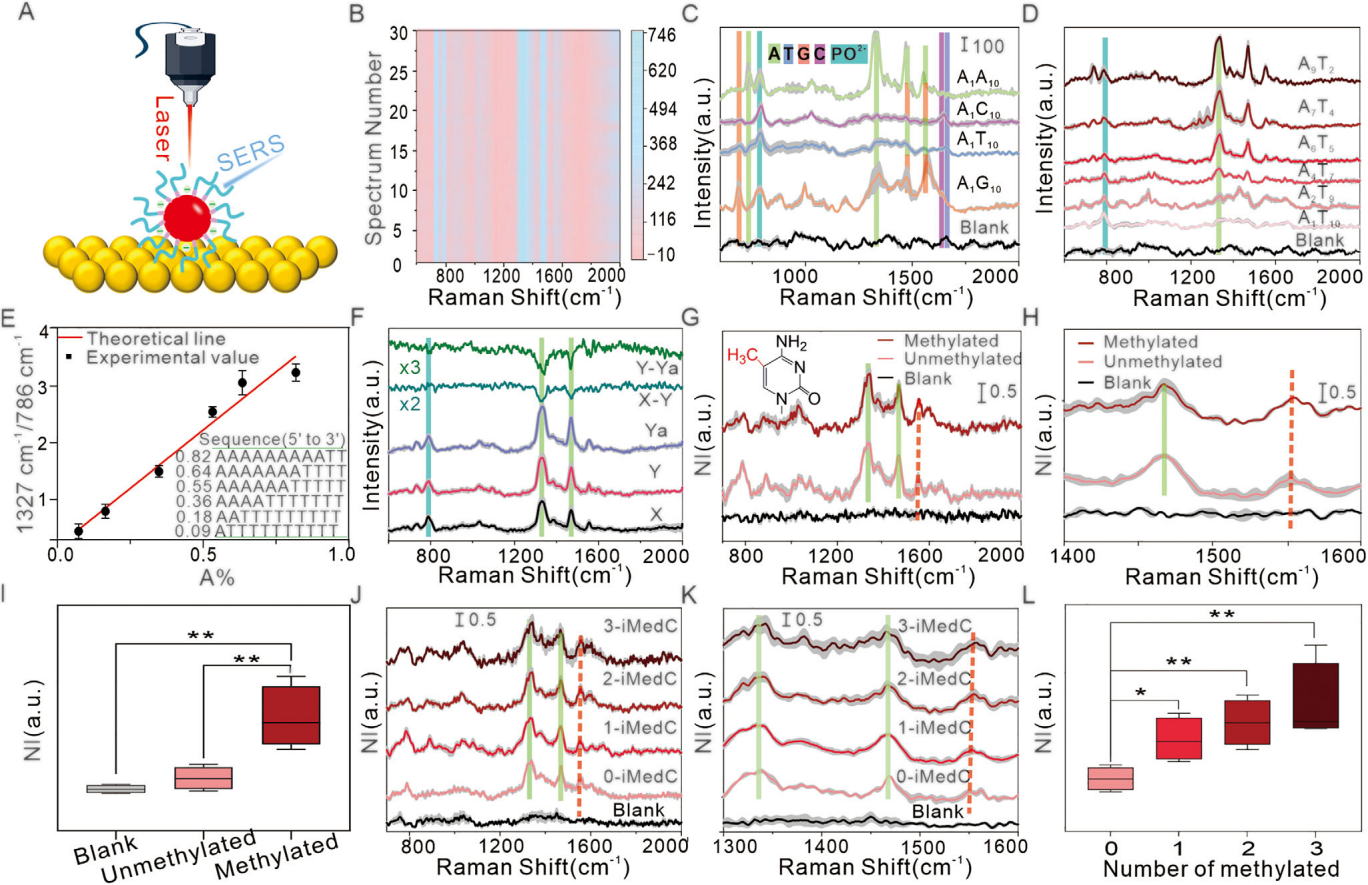
(A) Schematic illustration of SERS detection of DNA using SNAs. (B) Heatmap showing reproducibility of SERS signals from 30 independent experiments. (C) Assignment of characteristic Raman peaks for DNA bases. Frequency positions of characteristic nucleotide bands are highlighted in: green (adenine), blue (thymine), orange (guanine), purple (cytosine), and blue‐green (phosphoric acid). (D) SERS spectra of oligonucleotides containing varying numbers of A and T. (E) A plot of the relative peak intensity of 1327 cm^−1^/786 cm^−1^ with the real ratio value of A in these oligonucleotides. (F) SERS spectra of X, Y, and Ya are shown, together with the corresponding difference spectra (X‐Y and Y‐Ya) (G) SERS spectral of unmethylated and methylated DNA and blank. Inset: structural representation of 5‐methylcytosine. NI represents normalized intensity. (H) Methylation degree analysis SERS spectra and magnification band at 1400 to 1600 cm^−1^. (I) Raman intensity comparison between methylated and unmethylated DNA using one‐way ANOVA followed by Tukey's post‐hoc test (n = 10 per groups, ^*^ *p* < 0.05, ^**^ *p* < 0.01). (J) SERS spectra corresponding to different levels of methylation. (K) Magnified view of the SERS spectra corresponding to different methylation levels in the 1300–1600 cm^−1^. (L) Raman intensity comparison among graded DNA methylation levels (0, 1, 2, and 3 methylation sites) using one‐way ANOVA followed by Tukey's post‐hoc test. (n = 10 per groups, ^*^ *p* < 0.05, ^**^ *p* < 0.01).

The high sensitivity of DNA chemisorption to surface chemistry of nanoparticles may provide a scheme for label‐free SERS analysis of DNA‐featured structures, since the SERS technique is a sensitive fingerprint tool for surface molecular conformations [46, 47, 48, 49, 50, 51, 52, 53]. Here, our as‐synthesized SNAs were deposited on a silicon wafer to form a uniform SERS‐active array (Figure 6A). The signal reproducibility was evaluated by monitoring the intrinsic SERS signal of DNA (A_9_T_2_). SERS mapping based on the characteristic adenine band shows a uniform intensity distribution across the array (Figure 6B). This performance arises from the ordered, vertically aligned DNA layer and stable interparticle spacing, which generate a highly sensitive and reproducible SERS performance [45, 51, 54, 55, 56]. Adenine was chosen as a representative Raman marker owing to its strong and well‐resolved vibrational features [52]. To assign nucleotide‐specific vibrational features, 11‐mer DNA sequences (A_1_A_10_, A_1_C_10_, A_1_G_10_, and A_1_T_10_) were analyzed. All sequences exhibited a prominent PO_2_^−^ backbone vibration at 786 cm^−1^, while adenine, guanine, cytosine, and thymine showed distinct peaks at 1327, 675, 1631, and 1643 cm^−1^, respectively (Figure 6C, Table S2), consistent with previous reports [51, 52].

Nucleotide‐dependent distinct SERS features hint high molecular specificity, motivating us to explore how far this precise performance could be extended. SERS discrimination of nucleotide‐specific DNA structures is inherently challenging, as spectral intensities depend on molecular orientation and local plasmonic environments rather than solely on sequence composition [52, 57]. To mitigate these effects, the PO_2_^−^ backbone vibration was used as an internal standard, providing a stable reference that scales with nucleotide number for quantitative base‐composition analysis [52]. As examples, six 11‐mer oligonucleotides with varying A/(A+T) ratios were assessed (Figure 6D). Upon normalization to the PO_2_^−^ backbone vibration at 786 cm^−1^, subtle variations in adenine content were clearly resolved, as evidenced by the proportional increase of the 1327 cm^−1^ adenine peak. The relative Raman intensity ratio (1327/786 cm^−1^) exhibited a strong linear correlation with the A/(A+T) ratio (Figure 6E), confirming that PO_2_^−^ normalization enables quantitative comparison of base composition across sequences.

Three 11‐mer sequences, i.e., X (AAAAAAATTTT), Y (AAAAAAATTTA), and Ya (AAAAAAATTAT) were rationally designed to differ either by a single A‐T substitution at the 3′ terminus (X vs Y) or by a positional shift of an adenine toward the 5′ end while maintaining identical base composition (Ya vs Y). The SERS spectra of these three sequences were still dominated by adenine‐associated vibrational features, with subtle characteristic bands observable at 1327 and 1487 cm^−1^. To enhance these subtle differences, the spectra were normalized to the PO_2_^−^ backbone vibration, and difference spectra were obtained by subtracting Y from X (X‐Y) and Ya from Y (Y‐Ya). Strikingly, the difference spectra exhibited pronounced attenuation of the adenine‐associated vibrational bands at 1327 and 1487 cm^−1^. This intensity attenuation can be attributed either to the additional adenine in Y relative to X or to the closer proximity of an adenine base to plasmonic hot spots in Ya compared to Y, despite their identical adenine content (Figure 6F). These results demonstrate that this approach enables reliable discrimination of single‐nucleotide differences in DNA sequences, underscoring its potential for single‐nucleotide variant detection and precision DNA analysis.

This kind of SNAs could also give subtle information of DNA methylation in SERS analysis. SERS spectra displayed characteristic bands of adenine (1327 cm^−1^), the phosphate backbone (PO_2_^−^, 786 cm^−1^), and methyl groups (1552 cm^−1^, 5‐methylcytosines (i5MedC)) [58, 59]. Spectra were normalized to the adenine band, as adenine content was identical across samples, enabling reliable intensity comparison. The 1552 cm^−1^ band was assigned as a DNA methylation marker [58, 59] and exhibited pronounced intensity changes upon methylation, reflecting structural modifications (Figure 6G,H). SERS intensities of this band were systematically compared among blank, unmethylated, and methylated DNA using one‐way ANOVA with Tukey's post hoc test, revealing significantly higher intensity for methylated DNA (0.55) than unmethylated DNA (0.25, *p* < 0.01) (Figure 6I), confirming reliable discrimination of methylation states [59]. This strategy could also reveal the methylation density in DNA sequences. In detail, a series of DNA samples containing zero, one, two, and three i5MedC were assessed. Increasing methylation resulted in a progressive increase in the 1552 cm^−1^ intensity, which rose from approximately 0.26 for unmethylated DNA to 0.62 for DNA containing three i5MedC residues (*p* < 0.01) (Figure 6J,K). These findings establish highly sensitive, quantitative detection of DNA methylation through specific vibrational markers along with sequence‐specific and single‐base resolution capabilities.

## 3 Discussion

This study establishes a new interfacial paradigm for the synthesis of SNAs by leveraging AA as a weakly ionized, charge‐regulating ligand. In contrast to conventional approaches that rely on external environmental modulation, e.g., salt aging, low pH, freezing, or concentration methods [17, 18, 19, 20, 21, 23, 24, 25, 26, 60], this strategy fundamentally reconstructs the nanoparticle–ligand interface to intrinsically reduce the electrostatic repulsion between DNA and the gold surface. Such ligand‐mediated interfacial regulation represents a mechanistic departure from the long‐standing ionic screening model and introduces a more general, sequence‐universal route to SNAs construction under mild, physiologically relevant conditions.

Experimental analyses and MD simulations reveal that partially ionized AA^−^ ligands form a weakly charged interfacial layer. This weakly charged surface minimizes electrostatic repulsion between the DNA phosphate backbone and the gold core, while hydroxyl groups on AA^−^ establish transient hydrogen bonds with both nucleobases and phosphate moieties. These combined effects generate a synergistic electrostatic‐hydrogen‐bonding coupling mechanism, enabling spontaneous and energetically favorable DNA functionalization. Remarkably, even non‐thiolated DNA strands carrying a single terminal adenine can stably functionalize onto AA‐modified AuNPs, producing high‐density and structurally stable SNAs. This overcomes the limitations of conventional approaches, such as freezing‐assisted SNAs assembly requiring poly‐A tracts longer than ten bases (Figure S22) [17, 24, 26, 38], or microwave‐assisted methods that depend on terminal thymine residues [17, 23]. Although the References adsorption geometry of adenine on gold remains unresolved, the base‐substitution experiments strongly implicate the N6 exocyclic amino group in surface coordination. These findings suggest that thiol‐free DNA adsorption on AA‐AuNPs is governed not only by colloidal accessibility of the nanoparticle surface, but also by chemically selective nucleobase–gold interactions. In this context, the weakly screened interfacial environment established by AA may facilitate DNA access to the gold surface, thereby enabling effective adsorption of nonmodified DNA.

AA‐mediated SNAs exhibit upright DNA orientation and uniform interparticle gaps, enabling reproducible SERS. PO_2_^−^‐normalized spectra achieve single‐nucleotide resolution‐distinguishing both base identity and position, while the 1552 cm^−1^ marker quantitatively reports cytosine methylation levels. Beyond presynthesized AA‐AuNPs, the AA‐mediated ligand exchange for Cit, PVP, and CTAB‐stabilized colloids also demonstrates the success of this interfacial strategy, indicating a good generalization for broader materials and morphologies. AA represents a model for weakly ionic, hydrogen‐bond‐active ligands that bridge inorganic surfaces and biomolecular recognition motifs, suggesting a general interfacial framework where controlled surface charge governs function. By integrating charge modulation, hydrogen‐bond stabilization, and structural ordering, AA‐mediated SNAs combine high‐density loading, long‐term colloidal stability, and unparalleled SERS reproducibility, offering a chemically rational blueprint for label‐free, quantitative, and biocompatible nucleic acid detection.

Despite these advances, several important questions remain unresolved. Direct atomic‐scale characterization of the proposed interfacial coordination structure is still lacking. Furthermore, the proposed uniform DNA corona and plasmonic nanogap structures are currently supported mainly by indirect functional evidence rather than direct structural imaging. Finally, while the SNAs exhibit excellent colloidal stability across a broad pH range, more advanced surface‐sensitive techniques will be required to determine whether subtle irreversible interfacial chemical changes occur under extreme conditions. Addressing these questions in future studies will further improve the mechanistic understanding and structural characterization of non‐thiolated SNA systems.

## Author Contributions

**Zhonggang Liu**: conceptualization, methodology, software, formal analysis. **Cheng Wang**: conceptualization, methodology, software, data curation, visualization. **Guangping Li**: conceptualization, methodology, software, data curation, formal analysis, validation, investigation, writing – original draft, writing – review and editing. **Jinai Chen**: conceptualization, software. **Yu Zhang**: formal analysis, software, validation. **Zhongxiang Ding**: conceptualization, methodology, software, data curation. **Feng Wang**: conceptualization, writing – review and editing, formal analysis, validation. **Honglin Liu**: investigation, funding acquisition, writing – original draft, writing – review and editing, formal analysis, validation, supervision, resources, project administration, visualization. **Heng Gao**: conceptualization, methodology, software, data curation. **Xinyue Wu**: conceptualization, methodology, software, data curation, validation.

## Conflicts of Interest

The authors declare no conflicts of interest.

## Supporting information




**Supporting File 1**: advs75804‐sup‐0001‐SuppMat.docx.

**Supporting File 2**: advs75804‐sup‐0002‐MovieS1.mp4.

**Supporting File 3**: advs75804‐sup‐0003‐MovieS2.mp4.

**Supporting File 4**: advs75804‐sup‐0004‐MovieS3.mp4.

**Supporting File 5**: advs75804‐sup‐0005‐MovieS4.mp4.

## Data Availability

The data that support the findings of this study are available from the corresponding author upon reasonable request.
