# Supplementary material for: Ligand‐Defined Interfacial Chemistry Enables Instant and Sequence‐General DNA Chemisorption on Gold Nanoparticles Toward Label‐Free SERS With Single‐Base Resolution
Source: Adv Sci (Weinh). 2026 Jun 9:e75804. Online ahead of print. doi: 10.1002/advs.75804 (PMC13336010; doi:10.1002/advs.75804)
Supplement: Supplementary file 1 — Supporting File 1: advs75804‐sup‐0001‐SuppMat.docx. [file ADVS-9999-e75804-s004.docx]

Supporting Information
©Wiley-VCH 2021
69451 Weinheim, Germany

Ligand-Defined Interfacial Chemistry Enables Instant and Sequence-General DNA Chemisorption on Gold Nanoparticles Toward Label-Free SERS with Single-Base Resolution

Guangping Li,^#[a]^ Cheng Wang,^#[a]^ Xinyue Wu,^#[a]^ Zhongxiang Ding,^[b]^ Heng Gao,^[a]^ Zhonggang Liu,^[c]^ Yu Zhang,^[a]^ Jinai Chen,^[a]^ Feng Wang,^[a]^ Honglin Liu*^[a]^

Table of Contents

[Experimental Procedures 3](#_Toc220935966)

[Materials 3](#_Toc220935967)

[Instruments 3](#_Toc220935968)

[Preparation of AuNPs and AuNRs 3](#_Toc220935969)

[DNA-functionalization of AA-AuNPs 4](#_Toc220935970)

[Zeta potential and dynamic light scattering (DLS) measurements 4](#_Toc220935971)

[UV-vis absorption spectroscopy. 4](#_Toc220935972)

[Preparation of DNA-functionalized AuNPs via the salt-aging method 4](#_Toc220935973)

[Preparation of DNA-functionalization AuNPs by the freezing-based method 4](#_Toc220935974)

[Fluorescence quantification of the surface density of DNA@AA-AuNPs 5](#_Toc220935975)

[Surface ligand exchange on gold nanoparticles 5](#_Toc220935976)

[Molecular dynamic (MD) simulation 5](#_Toc220935977)

[SERS analysis on DNA fingerprint of single-base mutation 6](#_Toc220935978)

[Results and Discussion 7](#_Toc220935979)

[Supplementary Tables 7](#_Toc220935980)

[Supplementary Figures 10](#_Toc220935981)

[References 32](#_Toc220935982)

Experimental Procedures

**Materials**

Chloroauric acid (HAuCl_4_·4H_2_O, 99.99%) and L-ascorbic acid (AA) (C_6_O_8_H_6_, 99%) were obtained from Nanjing Chemical Reagent Co., Ltd. 2-Mercaptoethanol (ME), cetyltrimethylammonium bromide (CTAB), sodium borohydride (NaBH_4_), 5 bromosalicylic acid (BA), sodium dodecyl sulfate (SDS), sodium citrate dihydrate (Cit, C_6_H_5_Na_3_O_7_^.^2H_2_O, 99.0%), polyvinyl pyrrolidone (PVP) and butanol were supplied by Sinopharm Chemical Reagent Co., Ltd. NaCl, HCl, HNO_3_, and SDS were purchased from Aladdin (Shanghai, China). All other chemical reagents were of analytical grade. All other buffers and solutions were prepared using ultrapure water (18.25 MΩ, Millipore). DNA oligonucleotides were custom-synthesized by Sangon Biotech Co., Ltd. (Shanghai, China), and purified by HPLC.

**Instruments**

UV-vis analysis was carried out with a UV-vis Shimadzu UV-2600 spectrometer. Dynamic light scattering (DLS) size distribution was carried out with a Zetasizer instrument (Zetasizer Nano ZS Particle Sizer, ZEN3700, Malvern) at a temperature of 25°C. Fluorescence spectra were collected by a F98 fluoro spectrophotometer. Transmission electron microscopy (TEM) image was carried out on JEM1400FLASH TEM instrument.

**Preparation of AuNPs and AuNRs**

AuNPs were prepared using the ascorbic acid reduction method as previously reported.^1^ In brief, all the glass wares were soaked overnight in aqua regia (HNO_3_: HCl = 1:3), followed by rinsing with ultrapure water. AA was used as both the reducing agent and stabilizing ligand to synthesize AA-AuNPs with an average diameter of 23.2 ± 3.8 nm. Briefly, 50 mL of deionized water containing HAuCl₄ (0.25 mM) was added to a 100 mL round-bottom flask and heated to 80 °C under continuous stirring (500 rpm). Subsequently, 1 mL of 100 mM AA solution was rapidly injected into the reaction mixture at an approximate rate of 10 mL/s. The reaction was maintained at 80 °C with continuous stirring (500 rpm) for 8 min. After cooling to room temperature, the resulting deep-red solution was immediately centrifuged at 10 000 rpm for 10 min to remove residual reagents and then stored at 4 °C for further use. To prepare 48 nm AA-AuNPs, Deionized water (50 mL) containing HAuCl_4_ (3 mM) was added into a 100 mL round bottom flask. After heating to 90°C, 0.75 mL of 100 mM AA solution were rapidly added under vigorous stirring for 20 min. The deep red solution was cooled on ice and then centrifuged (6000 rpm, 10 min, and 4°C) to remove residual reagents.

AuNPs were prepared using the sodium citrate reduction method as previously reported.^2^ To prepare 13 nm AuNPs, 100 mL of 1 mM HAuCl_4_ solution was added into a 250 mL flask and then heated to boiling. Subsequently, 10 mL of 38.8 mM sodium citrate solution was added quickly with stirring, which resulted in a color change of the solution from pale yellow to wine red in 20 min. Then, stop heating the solution and cool it down to room temperature. Gold particles of about 25 nm were synthesized using a seed-mediated growth method.^3^

AuNRs were synthesized as described in a previous report.^4^ First, CTAB-capped Au clusters were prepared. A fresh ice-cold aqueous 10 mM NaBH_4_ solution (600 μL) was quickly added to 10 mL of an aqueous solution containing HAuCl_4_ (0.25 mM) and CTAB (100 mM) with vigorous stirring (350 rpm). The mixture was incubated at 30°C for 30 min to ensure the decomposition of NaBH_4_ in the mixture. This was the seed solution. To prepare the growth solution, a 4 mM AgNO_3_ solution (360 μL) was added to 5 mL of 100 mM CTAB solution containing 20 mM BA, and the solution was stirred (350 rpm) at 30°C for 7 min. Then, 5 mL of a 1 mM HAuCl_4_ solution was added. After 7 min of stirring, a 64 mM AA solution (40 μL) was added, and the solution was stirred for 30 s until it became colorless. Finally, the seed solution (16 μL) was added to the growth solution. The mixture was stirred for 30 s and left undisturbed at 30°C for 12 h. The final product was collected by centrifugation (13000 rpm, 7 min), washed twice with a 1 mM CTAB solution, and finally redispersed in 1 mL of 1 mM CTAB solution. The AuNRs solution was purified by centrifugation-driven depletion-induced flocculation. The AuNRs dispersed in 1 mL of a 1 mM CTAB solution were centrifuged. The resulting solution was centrifuged at 500 ×g for 5 min. After decanting the supernatant, the sediment was centrifuged, washed twice with 1 mM CTAB, once with 1% (w/v) SDS solution, and twice with 0.1% SDS solution, and finally dispersed in a 0.01% SDS solution for further use.

**DNA-functionalization of AA-AuNPs**

Vortex mixing: Thiolated or non-thiolated DNA was mixed with AA-AuNPs using a vortex mixer (SCILOGEX MX-S) at the maximum speed (~2000 rpm) for 10 s at room temperature (23 ± 3 °C). The resulting DNA-functionalized AA-AuNPs (DNA@AA-AuNPs) were purified by centrifugation at 10000 rpm for 15 min at room temperature to remove excess unbound DNA. After removal of the supernatant, the pellet was redispersed in ultrapure water or 0.5× TBE buffer (44.5 mM Tris, 44.5 mM boric acid, and 1 mM EDTA, pH 8.0). This washing procedure was repeated three times before further use.

Butanol dehydrating: The preparation process can be viewed in supplementary Movie 2. Briefly, Briefly, non-thiolated DNA and NaCl (final concentration: 5 mM) were added to the AA-AuNP solution at the desired DNA/AuNP molar ratio. In most experiments, 5 μL of 100 μM DNA was added to obtain a final DNA concentration of 5 μM. Subsequently, n-butanol was added at a volume ratio of 9:1 (n-butanol/AuNP solution), followed by vortex mixing (SCILOGEX MX-S, ~2000 rpm) for 10 s at room temperature (23 ± 3 °C). The resulting precipitate was redispersed in 0.5× TBE buffer using a volume equivalent to twice that of the original AuNP solution. The obtained DNA@AA-AuNPs were purified by centrifugation at 10000 rpm for 15 min at room temperature, followed by redispersion in ultrapure water or 0.5× TBE buffer. This washing procedure was repeated three times before further use.

**Zeta potential and dynamic light scattering (DLS) measurements**

The hydrodynamic size and zeta potential of citrate- and ascorbic acid–stabilized AuNPs and the corresponding SNAs were measured using a Zetasizer Nano-ZSE (Malvern Instruments). Prior to measurements, all AuNP and SNA samples were diluted 1,000-fold with nuclease-free water to minimize interparticle interactions and ensure an appropriate scattering intensity.

For DLS measurements, the diluted dispersions were transferred int cuvettes and equilibrated at 25°C for 2 min before analysis. Zeta potential was determined using folded capillary cells (DTS1070) under electrophoretic light scattering mode, and each value represents the average of at least three independent measurements.

**UV-vis absorption spectroscopy**

UV-vis absorption spectra of citrate/ascorbic acid-stabilized AuNPs and the corresponding SNAs were recorded using a UV-vis spectrophotometer (UV-2600). Prior to measurement, all samples were diluted with nuclease-free water to ensure that the absorbance fell within the linear detection range of the instrument.

Spectra were collected at room temperature using quartz cuvettes over a wavelength range of 200-800 nm, with nuclease-free water serving as the baseline reference.

**Preparation of DNA-functionalized AuNPs via the salt-aging method**

The preparation method for non-thiolated SNAs was carried out with reference to Fan’s work without modification.^5^ In brief, citrate-stabilized AuNPs (10 nM, 1000 μL) were first incubated with DNA (a final of 2 μM) at a molar ratio (DNA/AuNPs) of 200 for 16 h. The mixture was then brought to 10 mM sodium phosphate buffer (pH 7.4) and 0.1 M NaCl, and allowed to stand for 40 h. Afterward, the AuNPs were washed three times in 10 mM sodium phosphate buffer (pH 7.4) to remove excess DNA and resuspended with 0.5× TBE buffer until use.

**Preparation of DNA-functionalization AuNPs by the freezing-based method**

100 μL of the Cit-AuNP solution was mixed with 5 μL of 100 μM poly-A tagged DNA. The above mixture was frozen at -20°C for 1 h and then thawed at room temperature. After that, the particles were washed three times with 0.5× TBE buffer and resuspended for use.

**Fluorescence quantification of the surface density of DNA@AA-AuNPs**

Nonthiolated DNA used in these studies were labeled with FAM at 3′-terminal (Supplementary Table 1). FAM-labeled DNA were first absorbed to AA-AuNPs surface following the protocol as above described. The FAM-DNA-AuNPs were resuspended in 0.5× TBE buffer. The surface density of the DNA@AA-AuNPs was quantitated according to the published protocol.^6^ First, ME was added (20 mM final concentration) to the FAM-labeled DNA@AA-AuNPs solution and incubated overnight with shaking at room temperature. Then, the mixture was centrifugated at 15°C at 10000rpm for 20 min and the supernatant was collected. The released FAM-DNA probes were in the supernatant and the fluorescence was measured by F98 fluoro spectrophotometer. The fluorescence intensity was converted to the molar concentration of DNA probes by interpolation from a standard linear calibration curve that was prepared with known concentrations of same FAM-DNA probes at same buffer condition and ME concentration. AA-AuNPs concentration was determined via the absorption of UV-vis spectra and finally the number of DNA on each AA-AuNP could be calculated.

**Surface ligand exchange on gold nanoparticles**

Cit to AA; Citrate-coated gold nanospheres (Cit-AuNPs) were synthesized by a seed-mediated growth method previously reported. The obtained Cit-AuNPs (20 mL) were concentrated by centrifugation (8000 rpm, 10 min, 10 μL SDS (1%)), and the precipitates were dispersed into 20 mL Milli-Q water. The resulting solution was mixed with 10 μL SDS (1%), then the mixture was collected by centrifugation in the same condition. The precipitates were dispersed into 10 mL Milli-Q water. The resulting solution was mixed with 10 mL trisodium citrate (100 mM) 10 μL SDS (1%), then the mixture was collected by centrifugation in the same condition.

Cit to PVP; The obtained Cit-AuNPs (20 mL) were concentrated by centrifugation (8000 rpm, 10 min, 10 μL SDS (1%), 10 μL polysorbate 80 (0.01%)), and the precipitates were dispersed into 20 mL Milli-Q water. The resulting solution was mixed with 2 mL trisodium citrate (100 mM), 10 μL SDS (1%), and 10 μL polysorbate 80 (0.01%), then the mixture was collected by centrifugation in the same condition. The precipitates were re-dispersed in 2 mL PVP solution (1 wt %, dissolved in ethanol), the resulting solution was centrifuged at 7500 rpm for 20 min, then the collected PVP-AuNPs were re-dispersed in ethanol (2 mL) and stored for further use. By repeating the same procedure and replacing citrate with ascorbic acid, AA–AuNPs were obtained.

The CTAB-AuNRs suspensions were subjected to centrifugation at 6,500 g for 60 min. Nearly 95% of the supernatant was decanted, and the retentate was redispersed in 0.15 wt% Na-PSS to a final volume of 50 mL, and allowed to sit for at least 1 hour prior to the next step. In Stages 2 and 3, the AuNRs suspensions were subjected to centrifugation at 7,500 g for 30 min, then decanted from the supernatant and redispersed in 0.15 wt% Na-PSS to a final volume of 50 mL. Alternatively, PSS-AuNRs dispersions could be prepared at higher concentrations in 0.7 wt% Na-PSS, and diluted for later use. The PSS-AuNRs dispersions were stable at room temperature for at least several weeks. In Stage 4, PSS-AuNRs suspended in 0.15 wt% Na-PSS (30 mL) was centrifuged as described above in 15-mL plastic tubes. The supernatant was decanted until 0.2 mL of the retentate remained, and the PSS-AuNRs were redispersed into 30 mL of 5 mM ascorbic acid and allowed to sit for 12 hours. The AuNRs suspension was subjected to a second C/R cycle in the same manner, yielding 30 mL of GNRs dispersed in 5 mM ascorbic acid.

**Molecular dynamic (MD) simulation**

In this study, we employed *Materials Studio* to construct models of deprotonated ascorbic acid (AA⁻, one proton removed), citric acid (Cit³⁻, three protons removed), and gold nanoparticles (AuNPs) with a diameter of 10 nm. The molecular geometries of AA⁻ and Cit³⁻ were optimized using the *Gaussian 16* software packag, utilizing the B3LYP-D3 functional in conjunction with the 6-311G(d,p) basis set.^7^ The restrained electrostatic potential (RESP) charges were subsequently derived using *Multiwfn*.^8^ Force field parameters for gold nanoparticles were adopted from previously reported studies.^9-10^

All-atom molecular dynamics (MD) simulations were carried out using the *GROMACS* software package (version 2021.5).^11-12^ The Amber03 force field^13^ was employed to describe organic molecules, while the TIP3P water model^14^ was used for solvation. Initially, AuNPs were placed at the center of a simulation box, and 400 AA⁻ or Cit³⁻ molecules were randomly positioned within 1 nm of the AuNP surface using *Packmol*,^15^ thereby constructing AuNPs with two distinct surface modifications. These systems were then embedded in a cubic water box (14 nm × 14 nm × 14 nm) and electrically neutralized with Na⁺ counterions.

Subsequently, these modified AuNPs (AA-AuNP and Cit-AuNP) were introduced into a larger cubic simulation box (20 nm × 20 nm × 20 nm) containing 100 strands A₄T₇ oligonucleotides randomly distributed around the nanoparticle surfaces. Water molecules were added to solvate the systems, and NaCl was introduced to neutralize the overall charge and maintain a salt concentration of 5 mM.

Prior to production simulations, steepest descent energy minimization was performed to eliminate unfavorable contacts. This was followed by short (100 ps) simulations under both the NVT and NPT ensembles with position restraints applied to all heavy atoms except those in water, allowing complete equilibration of proteins and nanoparticles. The position restraints were then removed, and a 100 ns production run was conducted under the NPT ensemble. Pressure was maintained at 1.0 bar using a Parrinello–Rahman barostat, and temperature was held at 310 K using a velocity-rescale thermostat with a coupling constant τ = 0.1 ps. Non-bonded interactions were computed using a cutoff of 1.2 nm, and long-range electrostatic interactions were treated via the particle mesh Ewald (PME) method. All covalent bonds involving hydrogen atoms were constrained using the LINCS algorithm.^16^ Simulations were performed with a time step of 2 fs, and the neighbor list was updated every 10 steps. Periodic boundary conditions were applied in all three spatial dimensions.

**SERS analysis on DNA fingerprint of single-base mutation**

First, non-thiolated spherical nucleic acids SNAs were synthesized, followed by three washes with buffer solution. The SNA was transferred onto silicon/plasmonic substrates for SERS analysis. All Raman spectra were acquired and processed using BWSpec4 software, with a 785 nm laser at 50 mW power, 10,000 ms acquisition time, and one-time accumulation, followed by baseline subtraction.

Results and Discussion

Supplementary Tables

**Table S1.** Base sequences of DNA oligonucleotides employed in the present work.

| Names | Sequences (direction from 5' to 3') | Figure |
| --- | --- | --- |
| DNA21 | SH-AAAAAAAAACCCAGGTTCTCT | Figure 2A-E, 2F;  Figure S3-S4;  Figure 4F-H; |
| DNA21-FAM | SH-AAAAAAAAACCCAGGTTCTCT- FAM | Figure 2G; |
| A_1_-DNA | ACCCAGGTTCTCT | Figure 2F, I-K;  Figure4A-B, 4F-H;  Figure S6-S8, S10-S14, S19; |
| A_1_-DNA-FAM | ACCCAGGTTCTCT-FAM | Figure 2G, H, L;  Figure 4C;  Figure S9 |
| A_2_-DNA-FAM | AACCCAGGTTCTCT-FAM | Figure 2G, 2L;  Figure S9 |
| A_3_-DNA-FAM | AAACCCAGGTTCTCT-FAM |  |
| A_4_-DNA-FAM | AAAACCCAGGTTCTCT-FAM |  |
| A_6_-DNA-FAM | AAAAAACCCAGGTTCTCT-FAM |  |
| A_9_-DNA-FAM | AAAAAAAAACCCAGGTTCTCT-FAM |  |
| A_12_-DNA-FAM | AAAAAAAAAAAACCCAGGTTCTCT-FAM |  |
| DNA-a1 | AAAAATTTTTCGACTTAT | Figure 3A |
| DNA-a2 | CGACTTCAAAAATTTTTT |  |
| DNA-a3 | CGACTTATTTTTTAAAAA |  |
| A_5_-DNA | AAAAACCCAGGTTCTCT |  |
| A_5_-_1_A DNA | AAAAACCCAGGATTCTCT |  |
| A_5_-_3_A DNA | AAAAACCCAGGAAATTCTCT |  |
| A_5_-_5_A DNA | AAAAACCCAGGAAAAATTCTCT |  |
| A_5_-A_1_ DNA | AAAAACCCAGGTTCTCTA |  |
| A_5_-A_3_ DNA | AAAAACCCAGGTTCTCTAAA |  |
| A_5_-A_5_ DNA | AAAAACCCAGGTTCTCTAAAAA |  |
| A_5_-A_3_T_1_ DNA | AAAAACCCAGGTTCTCTAAAT |  |
| A_5_-A_3_T_2_ DNA | AAAAACCCAGGTTCTCTAAATT |  |
| A_5_-A_3_T_3_ DNA | AAAAACCCAGGTTCTCTAAATTT |  |
| A_1_-1-DNA | ACCCAGGTTCTCT |  |
| A_2_-1-DNA | AACCCAGGTTCTC |  |
| A_3_-1-DNA | AAACCCAGGTTCTC |  |
| A_4_-1-DNA | AAAACCCAGGTTCTC |  |
| A_6_-1-DNA | AAAAAACCCAGGTTCTC |  |
| A_9_-1-DNA | AAAAAAAAACCCAGGTTCTCT |  |
| A_12_-1-DNA | AAAAAAAAAAAACCCAGGTTCTC |  |
| A_1_T_10_ | ATTTTTTTTTT | Figure 3C;  Figure 6C, D;  Figure S18 |
| I_1_T_10_ | ITTTTTTTTTT | Figure 3C |
| C_1_T_10_ | CTTTTTTTTTT | Figure S18 |
| G_1_T_10_ | GTTTTTTTTTT |  |
| T_1_T_10_ | TTTTTTTTTTT |  |
| A_11_ | AAAAAAAAAAA | Figure 4E;  Figure 6C |
| A_5_T_6_ | AAAAATTTTTT | Figure 4E;  Figure 6D; |
| T_6_A_5_ | TTTTTTAAAAA | Figure 4E; |
| 5AT_6_ | TTTAAAAATTT |  |
| A_1_C_10_ | ACCCCCCCCCC | Figure 6C |
| A_1_G_10_ | AGGGGGGGGGG |  |
| A_2_T_9_ | AATTTTTTTTT | Figure 6D |
| A_4_T_7_ | AAAATTTTTTT |  |
| A_7_T_4_ | AAAAAAATTTT |  |
| A_9_T_2_ | AAAAAAAAATT |  |
| X | AAAAAAATTTT | Figure 6F |
| Y | AAAAAAATTTA |  |
| Ya | AAAAAAATTAT |  |
| CH_3_C-0 | AAAACCCTTTT | Figure 6G-L |
| CH_3_C-1 | AAAAA/i5MedC/CCTTTT |  |
| CH_3_C-2 | AAAAA/i5MedC//i5MedC/CTTTT |  |
| CH_3_C-3 | AAAAA/i5MedC//i5MedC//i5MedC/TTTT |  |
| A_9_-DNA | AAAAAAAAACCCAGGTTCTCT | Figure S15-S16 |

**Table S2.** Raman frequencies of typical vibrational modes of DNA.

| Raman shift (cm^-1^) | SERS band assignments | ref |
| --- | --- | --- |
| 675 | G, ring breathing | ^17-19^ |
| 723 | A | ^17^ |
| 786 | PO^2−^, skeleton stretching | ^17^ |
| 1327 | A | ^17-19^ |
| 1487 | A, G | ^18-19^ |
| 1552 | CH_3_ | ^20^ |
| 1570 | A, G | ^18-19^ |
| 1631 | T | ^17^ |
| 1643 | C | ^17^ |

Supplementary Figures


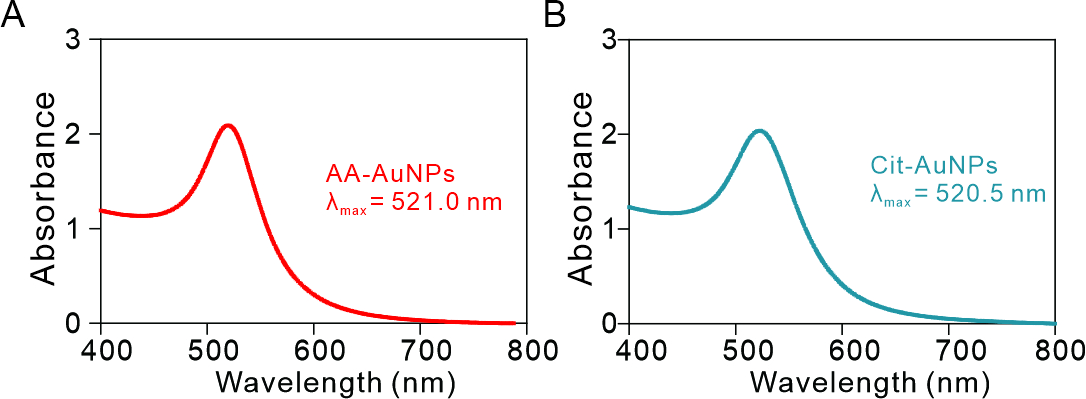


**Figure S1.** Characterizations of AA-AuNPs and Cit-AuNPs. The absorption spectra of AA-AuNPs **(A)** and the Cit -AuNPs **(B)**.


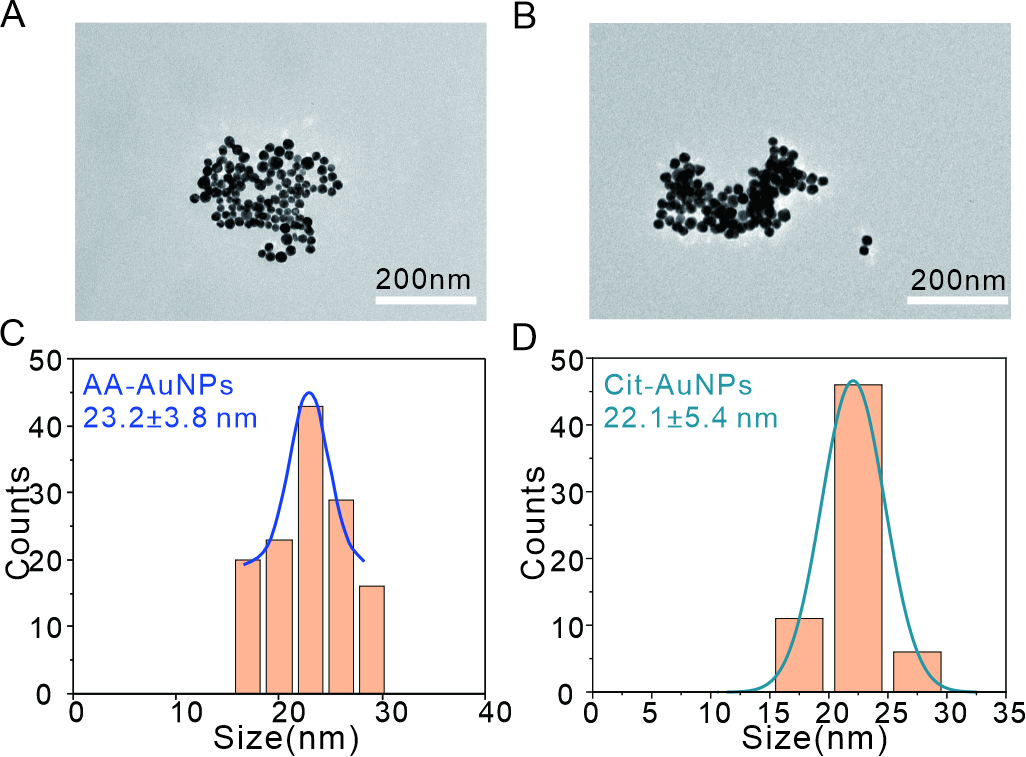


**Figure S2.** Characterization of AA-AuNPs and Cit-AuNPs. TEM images of AA-AuNPs **(A)** and Cit-AuNPs **(B)**. Size distribution profiles of AA-AuNPs **(C)** and Cit-AuNPs **(D)** determined from TEM measurements.


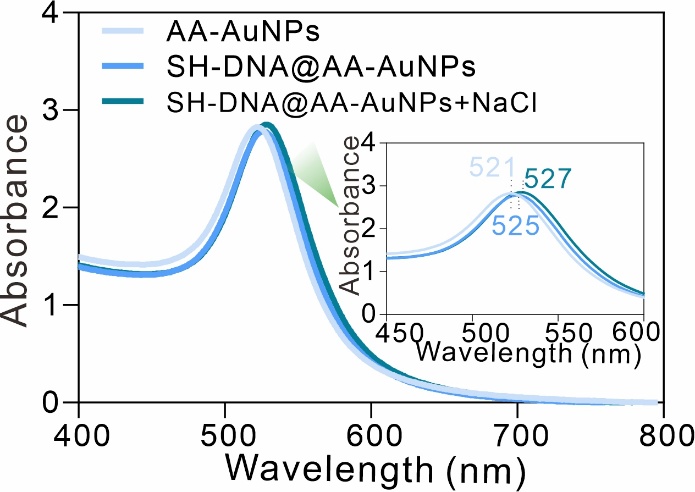


**Figure S3.** UV-vis absorption spectra of SH-DNA vortex-mixed with AA-AuNPs under salt-free and 5 mM salt-containing conditions. The inset shows a magnified view of the 450-600 nm region.


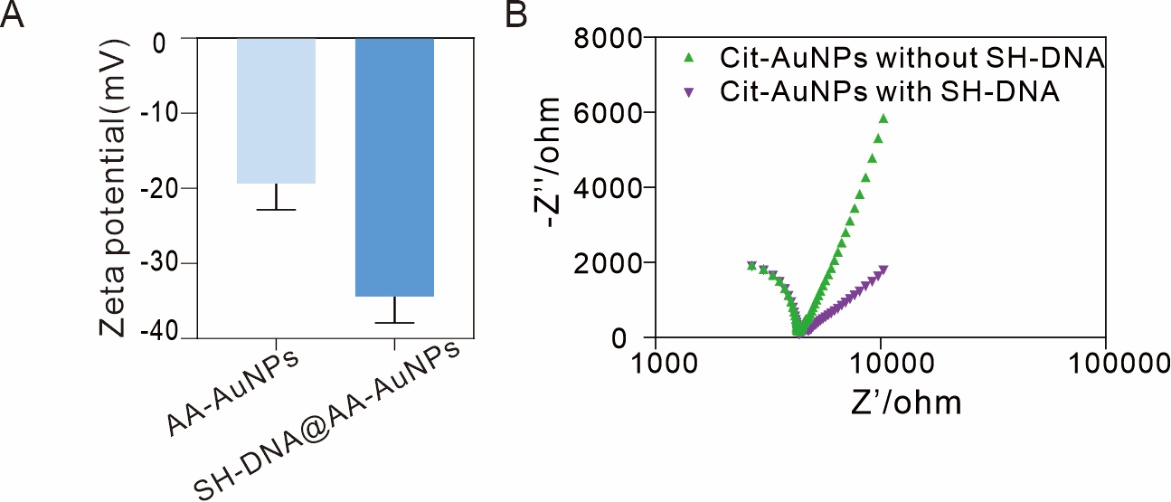


**Figure S4.** **(A)** Zeta potentials of bare AA-AuNPs and SH-DNA@AA-AuNPs. Error bars represent the standard deviation from three independent measurements. **(B)** Nyquist plots of AA-AuNPs surfaces before and after SH-DNA addition, recorded using 5 mM $[Fe(CN)_{6}]^{3-/4-}$as the redox mediator.


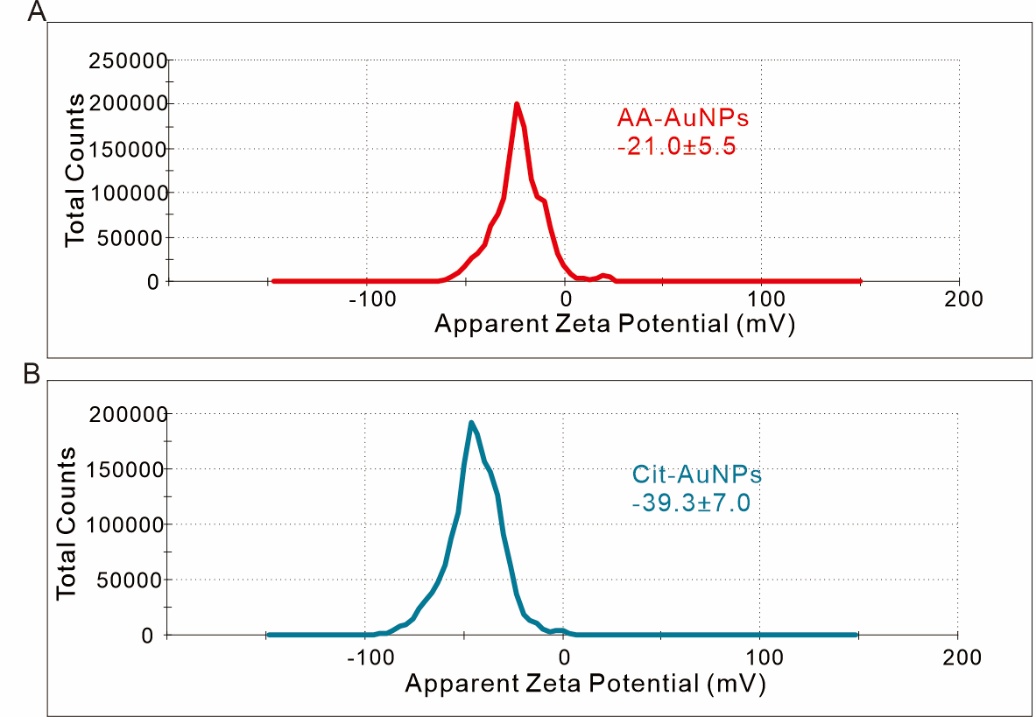


**Figure S5.** Characterizations of AA-AuNPs and Cit-AuNPs. The zeta-potential distribution profiles of the AA-AuNPs **(A)** and the Cit-AuNPs **(B)**.


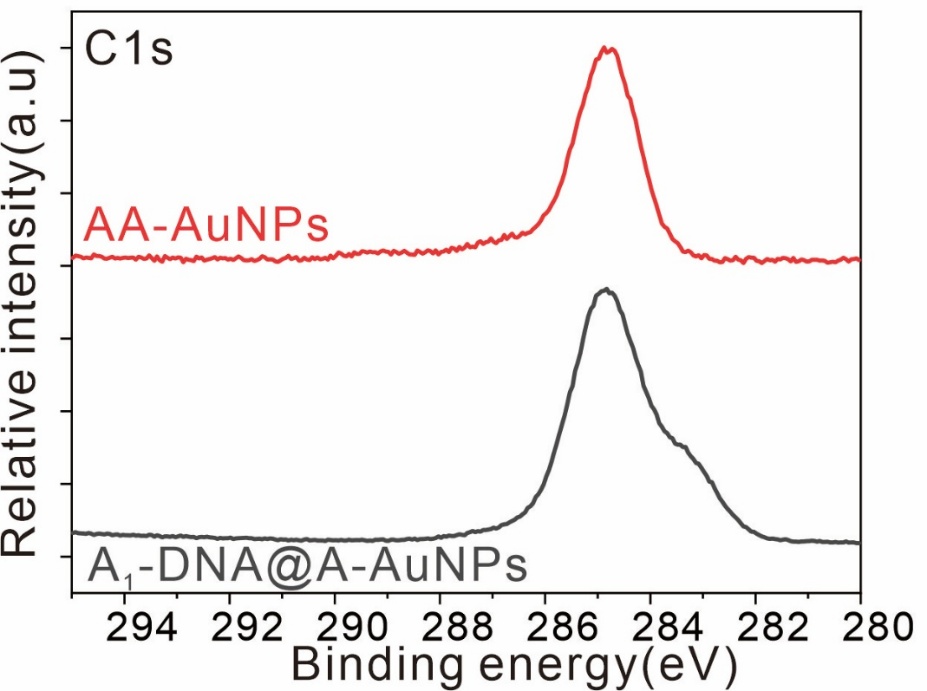


**Figure S6.** The C1s XPS binding energy profiles of AA-AuNPs, the A_1_-DNA@AA-AuNPs.


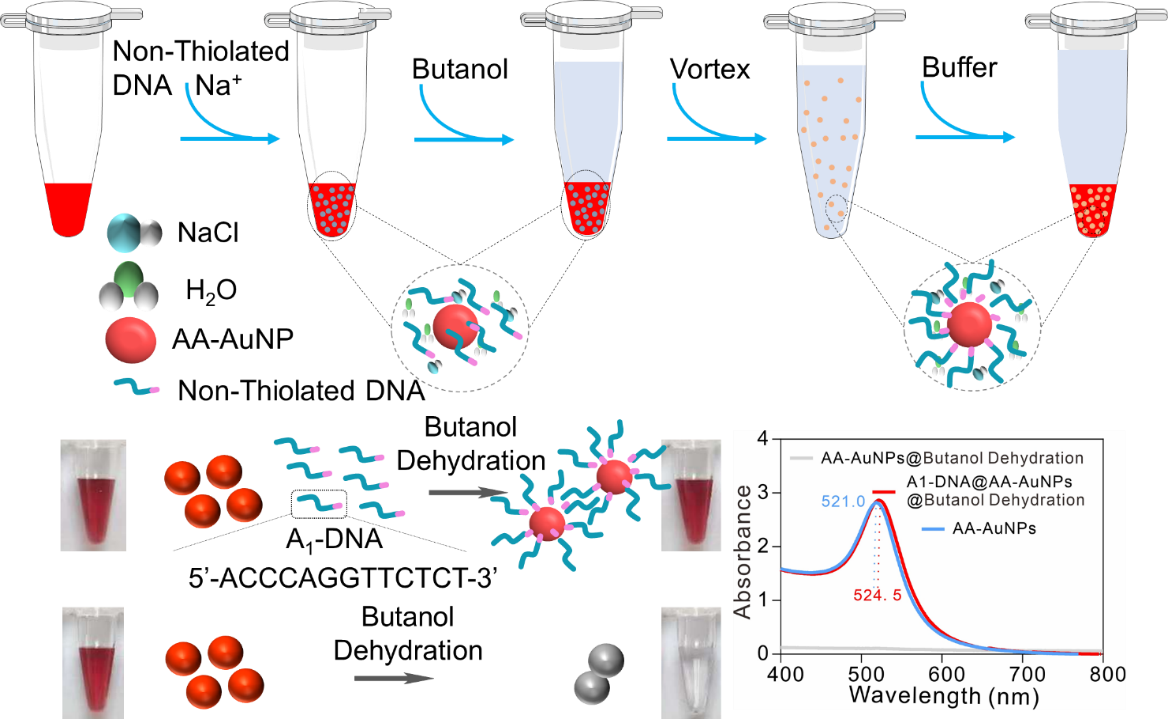


**Figure S7.** Scheme illustrating the attachment of non-thiolated DNA to AA-AuNPs *via* the butanol dehydration. UV-vis absorption spectra confirming the formation of DNA-functionalized AA-AuNPs.


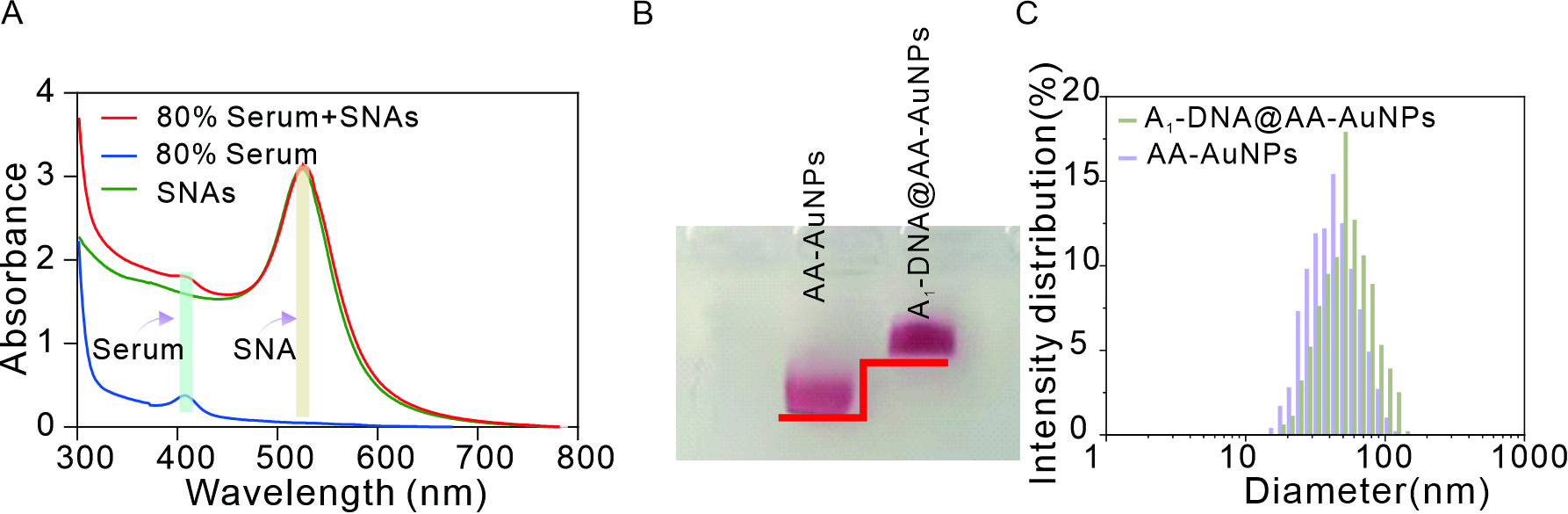


**Figure S8.** **(A)** UV–vis absorption spectra of freshly prepared SNAs and SNAs after incubation in 80% serum. **(B)** Agarose gel electrophoresis of samples with and without A_1_-DNA *via* the butanol dehydration. **(C)** Dynamic light scattering measurements of the hydrodynamic diameters of bare AA-AuNPs and A_1_-DNA@AA-AuNP.


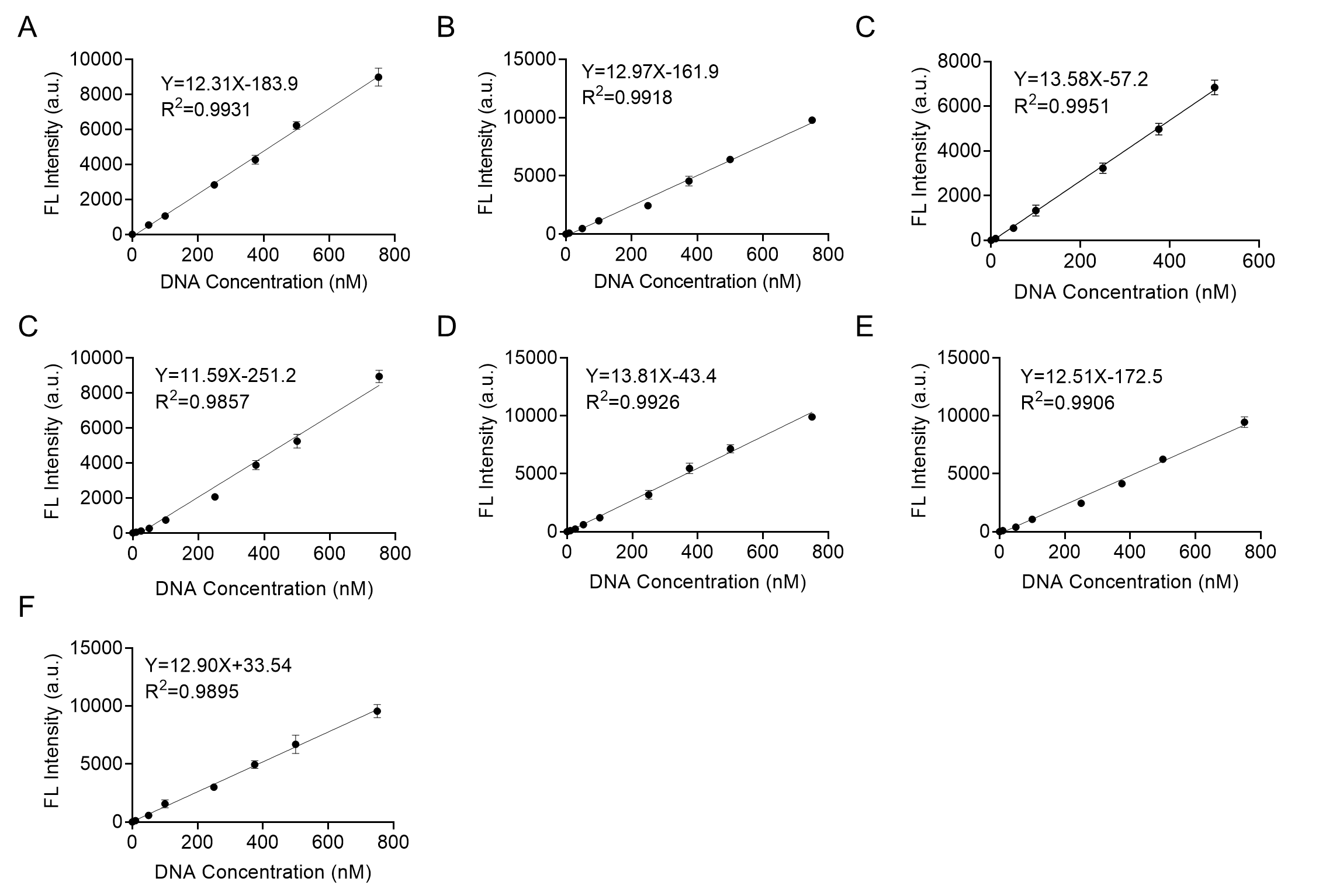


**Figure S9.** Fluorescence calibration curve of the corresponding DNA. **(A)** A_1_-DNA-FAM. **(B)** A_2_-DNA-FAM. **(C**) A_3_-DNA-FAM. **(D)** A_4_-DNA-FAM. **(E)** A_6_-DNA-FAM. **(F)** A_9_-DNA-FAM. **(G)** A_12_-DNA-FAM. Error bars represent standard deviation from three independent tests.


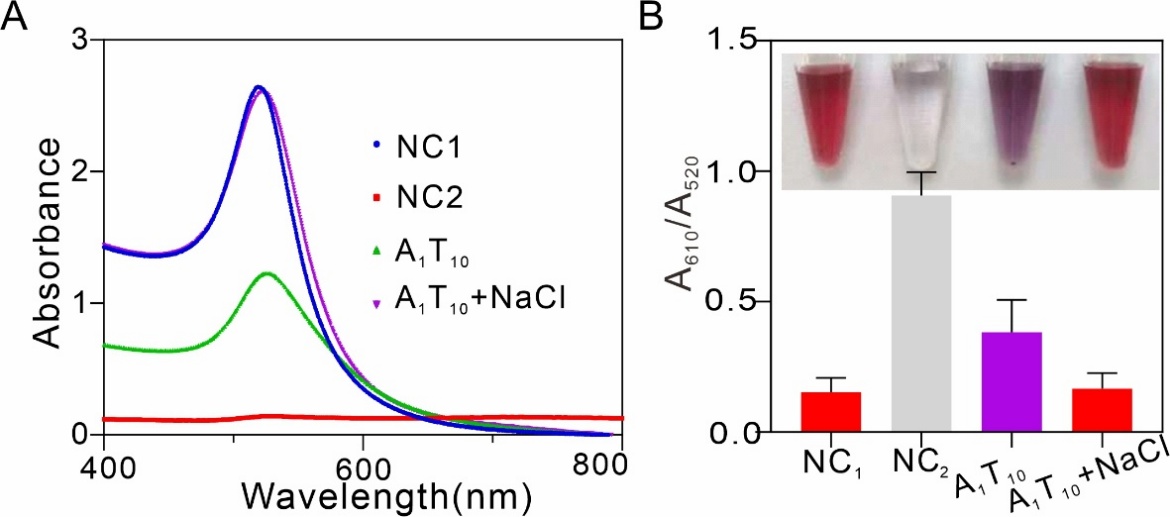


**Figure S10.** **(A)** Effect of 5 mM NaCl on A_1_T_10_ functionalization AA-AuNPs. **(B)** UV-vis A₆₁₀/A₅₂₀ ratios of SNAs synthesized *via* the butanol dehydration under salt-free and salt-present conditions. The inset shows photographs of the corresponding sol states. NC1: Negative control negative control without the butanol dehydration or A_1_T_10_; NC2: negative control with the butanol dehydration but without A_1_T_10_. Error bars represent standard deviation from three independent tests.


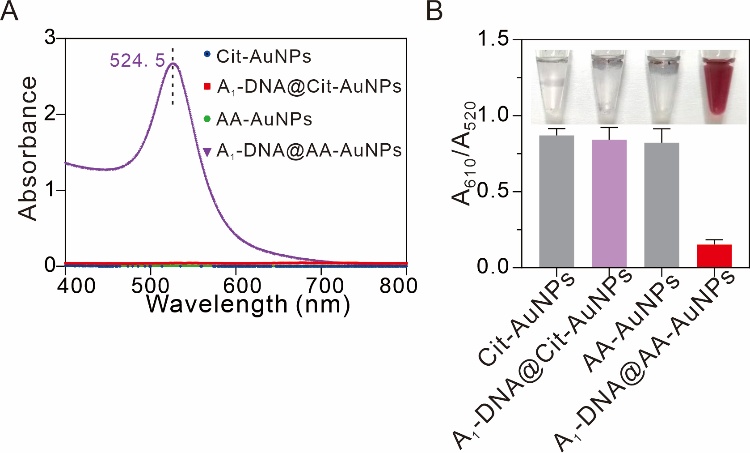


**Figure S11.** **(A)** UV-vis absorption spectra of A_1_-DNA -functionalized Cit/AA-AuNPs prepared *via* the butanol dehydration. **(B)** UV-vis A₆₁₀/A₅₂₀ ratios of A_1_-DNA functionalized Cit/AA-AuNPs. The inset shows photographs of the corresponding SNA sol states. Error bars represent the standard deviation from three independent measurements.


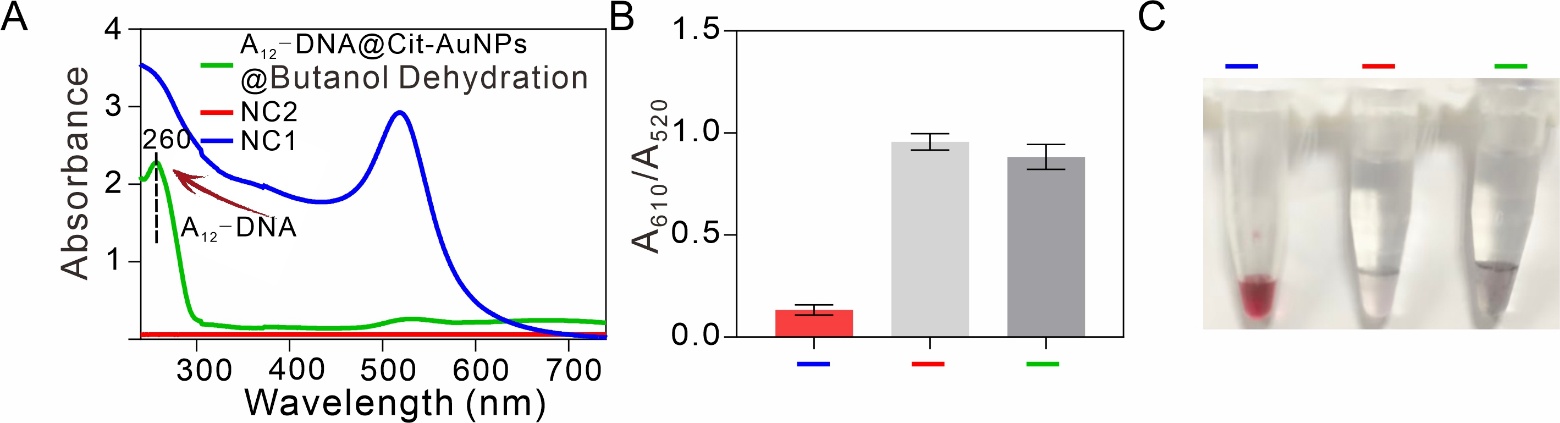


**Figure S12.** **(A)** UV-vis absorption spectra of citrate-capped AuNPs prepared under the conditions described *via* the butanol dehydration. **(B)** UV-vis A₆₁₀/A₅₂₀ ratios of A_12_-DNA-functionalized Cit-AuNPs. Error bars represent the standard deviation of three independent measurements. **(C)**The inset shows photographs of the corresponding sol states.


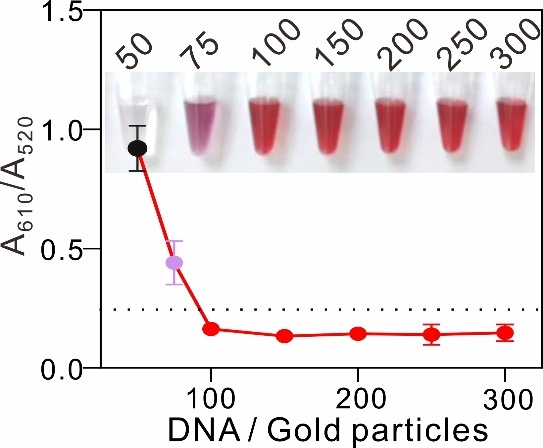


**Figure S13.** Effect of the A_1_-DNA@AA-AuNP molar ratio on DNA functionalization *via* the butanol dehydration. Error bars represent the mean ± SD for three independent replicates.


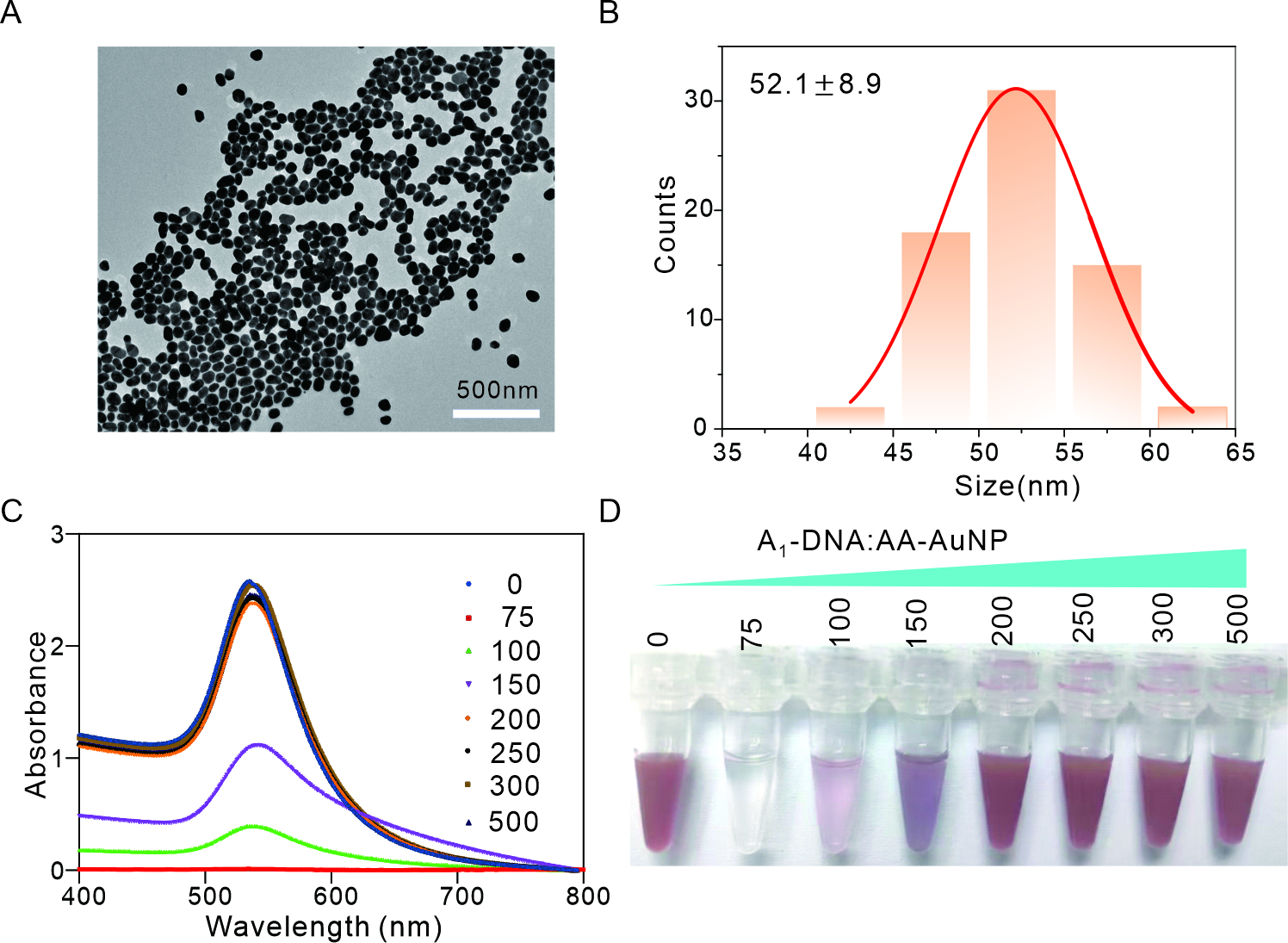


**Figure S14.** Effect of the A_1_-DNA@AA-AuNP molar ratio on DNA functionalization *via* the butanol dehydration method. **(A)** TEM image of AA-AuNPs. **(B**) Size distribution of AA-AuNPs determined from TEM analysis. (C) UV-vis absorption spectra of AA-AuNPs mixed with DNA at varying DNA/AuNP molar ratios. (D) Photographs of the corresponding samples prepared at different DNA/AuNP molar ratios.


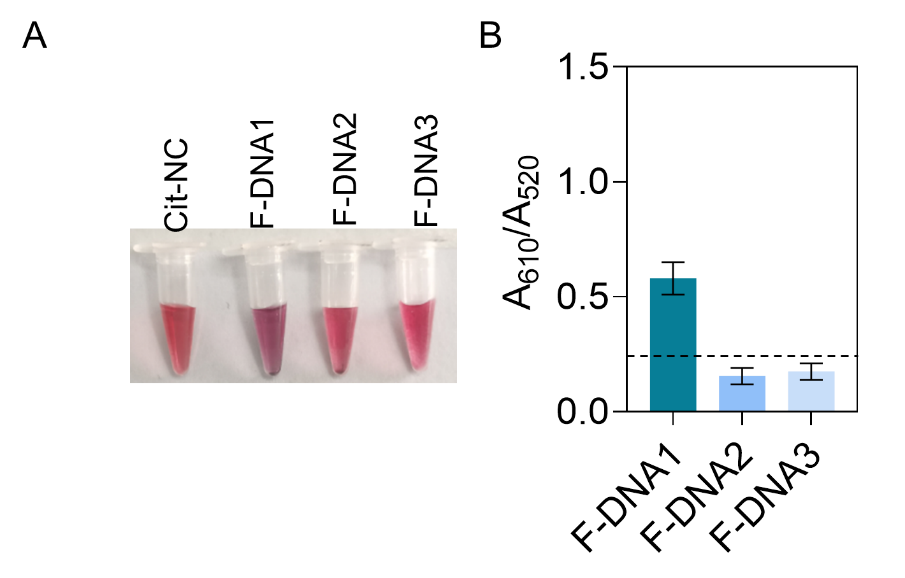


**Figure S15.** Different DNA-functionalized Cit–AuNPs *via* the freezing method. **(A)** Photographs of SNAs formed with three different DNA sequences. **(B)** UV-vis A₆₁₀/A₅₂₀ ratios of the SNAs. Error bars represent the mean ± SD from three independent experiments.


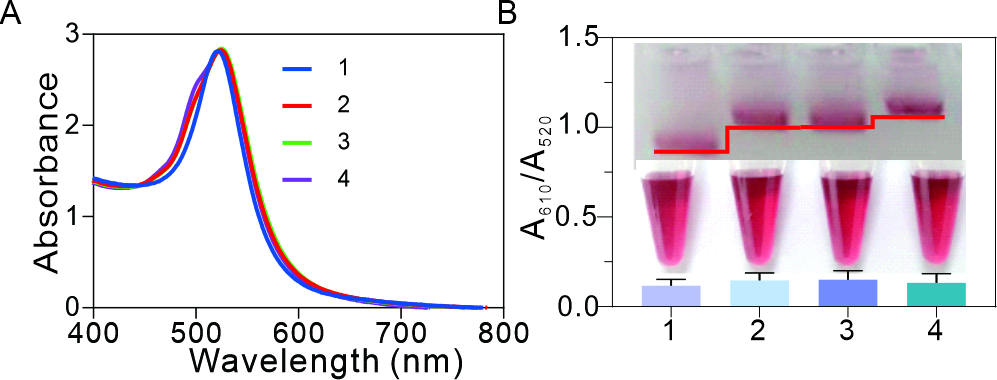


**Figure S16.** **(A)** The UV–vis absorption spectra of three different DNA-functionalized AA-AuNPs *via* the butanol dehydration method. **(B)** The UV-vis A_610_/A_520_ ratio with three different DNA-functionalized AA-AuNPs. The inset illustrates the photograph shows the colloid states and agarose gel electrophoresis of the three different DNA functionalization AA-AuNPs. The numbers 1, 2, 3, and 4 are represented by AA-AuNPs, A_9_-DNA@AA-AuNPs, A_10_-DNA1@AA-AuNPs, and A_10_-DNA2@AA-AuNPs, respectively.

**Figure S17.** The amounts of varied DNA strands on AA-AuNPs, synthesized *via* the butanol dehydration method at a fixed DNA/AA-AuNP molar ratio of 500:1. Error bars represent standard deviation from three independent tests.


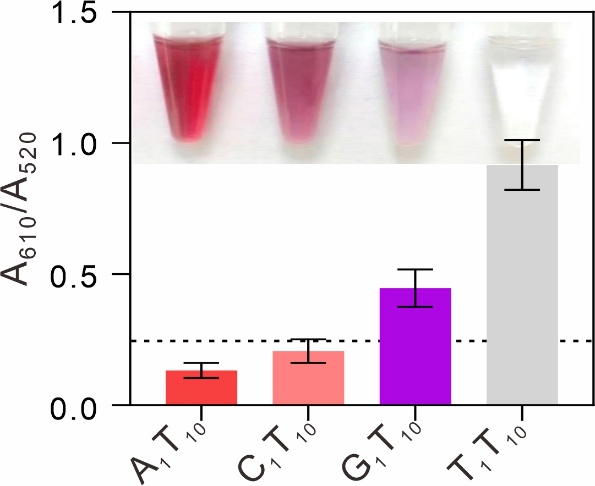


**Figure S18.** Stability of DNA@AA-AuNPs prepared with X_1_T_10_ (X = A, C, G, T) *via* the butanol dehydration method. Error bars represent the mean ± SD for three independent replicates.

**
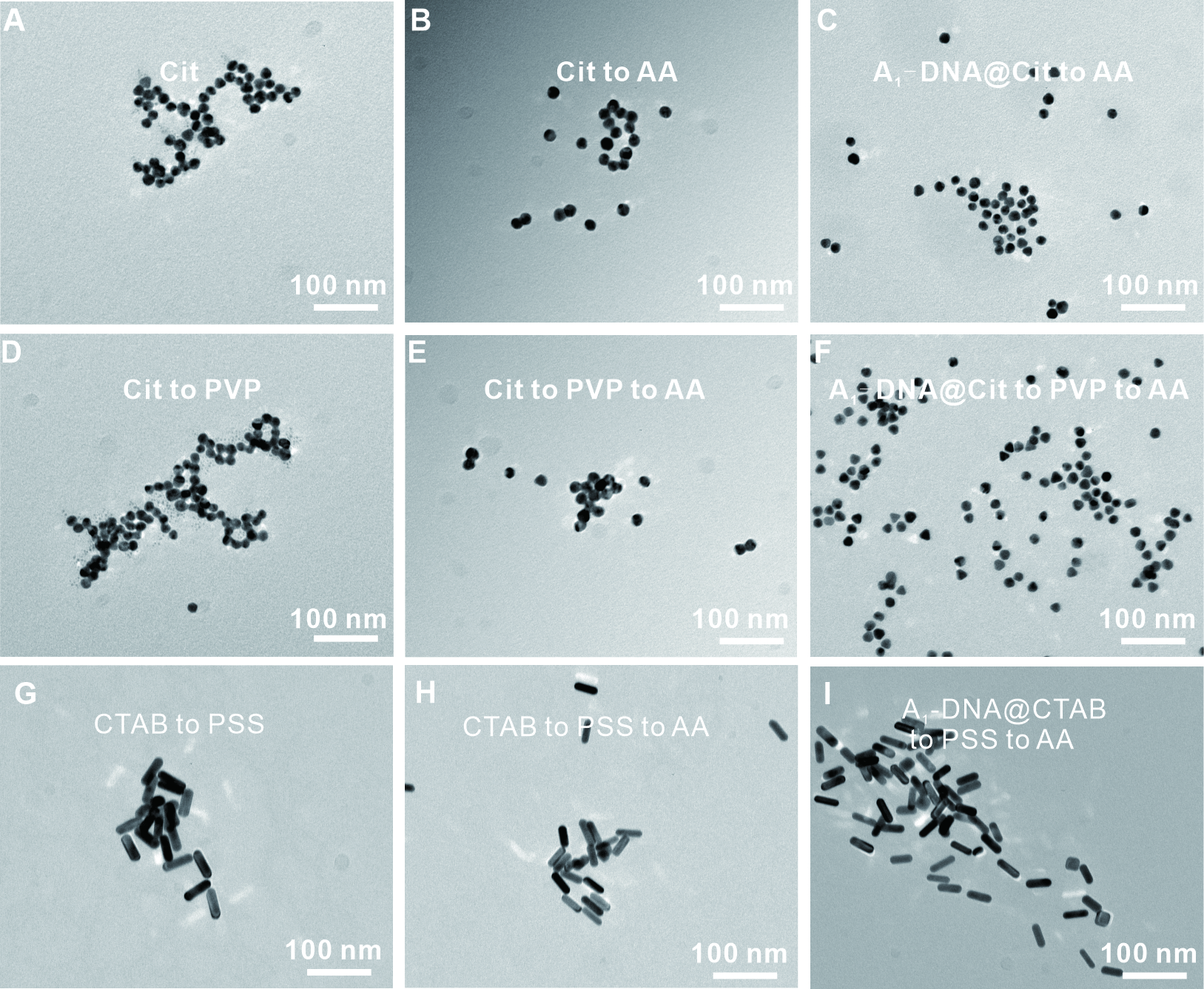
**

**Figure S19.** Stability and DNA-functionalization capability of gold nanostructures with different surface ligands. **(A)** TEM image of gold nanoparticles stabilized with sodium citrate. **(B)** TEM image of gold nanoparticles after ligand exchange from sodium citrate to ascorbic acid (AA). **(C)** TEM image of DNA-functionalized gold nanoparticles following citrate-to-AA ligand exchange. **(D, E)** TEM images of gold nanoparticles functionalized with PVP and subsequently exchanged to AA. **(F)** TEM image of DNA-functionalized gold nanoparticles after PVP-to-AA ligand exchange. **(G**) TEM image of gold nanorods after ligand exchange from CTAB to PSS. **(H)** TEM image of gold nanorods after subsequent ligand exchange from PSS to AA. **(I)** TEM image of DNA-functionalized gold nanorods following PSS-to-AA ligand exchange.


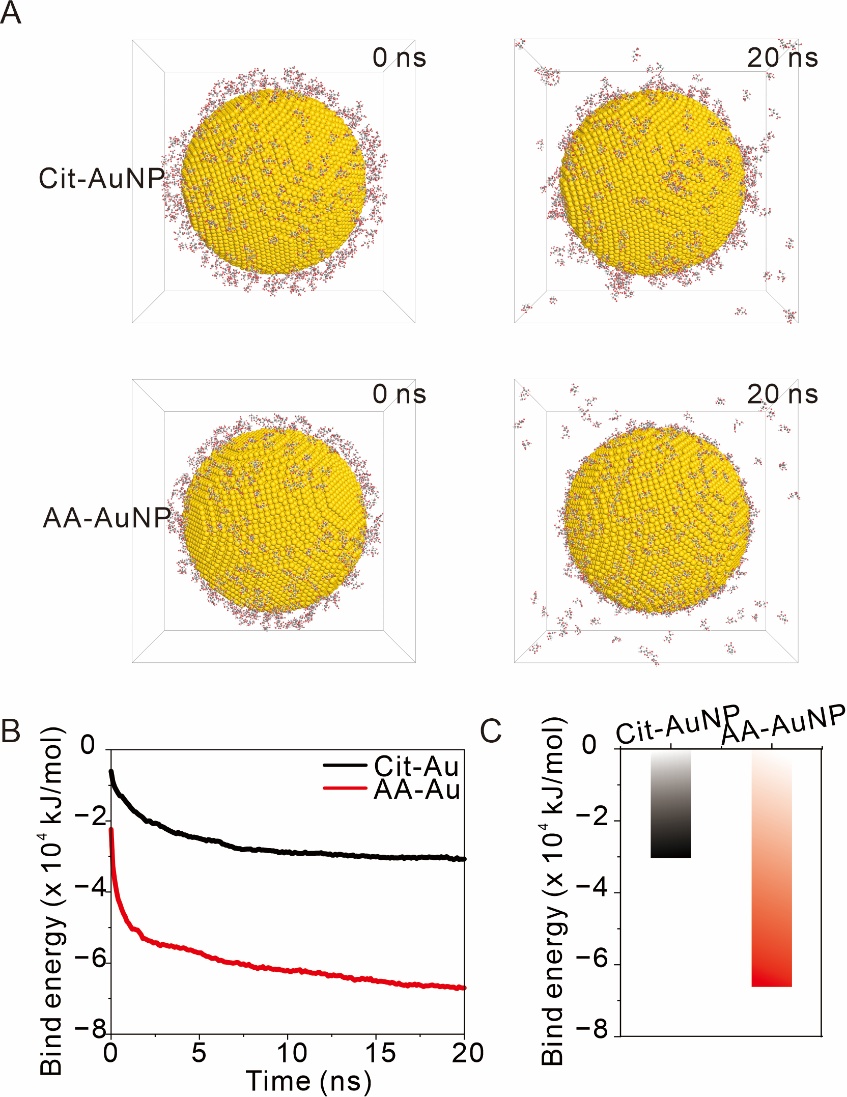


**Figure S20**. Theoretical studies of AA-Au and Cit-Au surfaces. **(A)** Representative snapshots from molecular dynamics (MD) simulations of Cit³⁻ and AA⁻ on Au surfaces (~20 × 20 nm²) at 0 ns and 20 ns. Initially, 400 Cit³⁻ or AA⁻ molecules were loaded at pH 7.5 (surface density ~1.3/nm²), with sufficient Na⁺ added to neutralize the charges. Water molecules and nonpolar H atoms are omitted for clarity. After 20 ns, 57 Cit³⁻ and 54 AA⁻ molecules detached from the Au surface and were removed in subsequent simulations. **(B)** Time-dependent total binding energy profiles of the Au surface with Cit³⁻ or AA⁻ during MD simulations. **(C)** Average binding energy of the Au surface with Cit³⁻ or AA⁻ over the last 30 ns of simulatio.


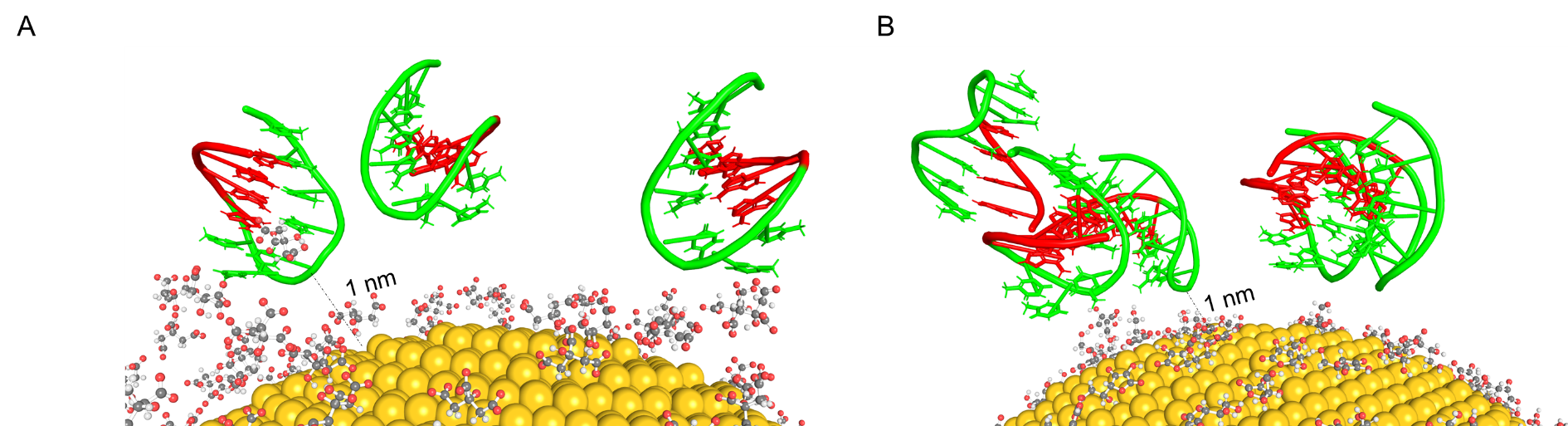


**Figure S21**. Initial DNA–AuNP distances in molecular dynamics simulations with different surface ligands: **(A)** Cit-AuNPs; **(B)** AA-AuNPs.


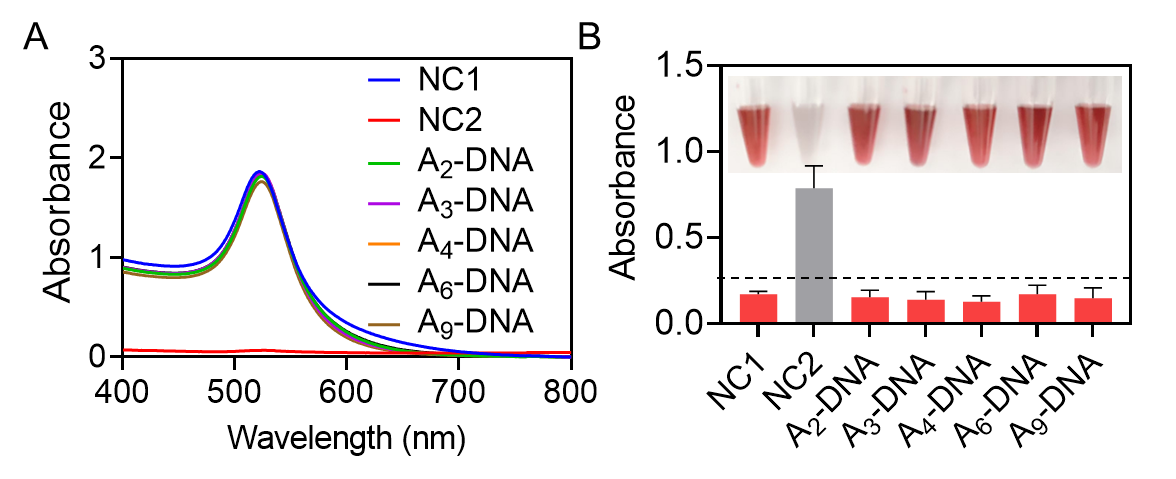


**Figure S22.** Freezing-mediated synthesis of spherical nucleic acids (SNAs) using DNA with poly-A blocks of varying lengths. **(A)** UV-vis absorption spectra of AA–AuNPs functionalized with poly-A block DNA of different lengths via the freezing method. **(B)** UV-vis A₆₁₀/A₅₂₀ ratios corresponding to different poly-A block lengths. The inset shows photographs of the colloidal states of the prepared SNAs. NC1: negative control consisting of bare AuNPs (no DNA and no freezing treatment). NC2: negative control consisting of AuNPs without DNA but subjected to the freezing treatment. Error bars represent the mean ± SD from three independent experiments.

# References

1. Zhang, J.; Huang, Z.; Xie, Y.; Jiang, X., Modulating the catalytic activity of gold nanoparticles using amine-terminated ligands. *Chem Sci* **2022,** *13* (4), 1080-1087.

2. Wang, X.; Yang, Z.; Li, Z.; Huang, K.; Cheng, N.; Liu, J., Rapid Thermal Drying Synthesis of Nonthiolated Spherical Nucleic Acids with Stability Rivaling Thiolated DNA. *Angew Chem Int Ed* **2024,** *63* (49), e202410353.

3. Ding, Z.; Gao, H.; Wang, C.; Li, Y.; Li, N.; Chu, L.; Chen, H.; Xie, H.; Su, M.; Liu, H., Acoustic Levitation Synthesis of Ultrahigh-Density Spherical Nucleic Acid Architectures for Specific SERS Analysis. *Angew Chem Int Ed Engl* **2024,** *63* (20), e202317463.

4. Kim, J.-M.; Kim, J.; Choi, K.; Nam, J.-M., Plasmonic Dual-Gap Nanodumbbells for Label-Free On-Particle Raman DNA Assays. *Adv. Mater* **2023,** *35* (15), 2208250.

5. Pei, H.; Li, F.; Wan, Y.; Wei, M.; Liu, H.; Su, Y.; Chen, N.; Huang, Q.; Fan, C., Designed Diblock Oligonucleotide for the Synthesis of Spatially Isolated and Highly Hybridizable Functionalization of DNA–Gold Nanoparticle Nanoconjugates. *Journal of the American Chemical Society* **2012,** *134* (29), 11876-11879.

6. Huang, M.; Xiong, E.; Wang, Y.; Hu, M.; Yue, H.; Tian, T.; Zhu, D.; Liu, H.; Zhou, X., Fast microwave heating-based one-step synthesis of DNA and RNA modified gold nanoparticles. *Nature Communications* **2022,** *13* (1).

7. Petersson, G. A. a. M. A. A. L., A complete basis set model chemistry. II. Open‐shell systems and the total energies of the first‐row atoms. *The Journal of Chemical Physics* **1991,** *94(9): 6081-6090.*

8. Lu, T.; Chen, F., Multiwfn: A multifunctional wavefunction analyzer. *Journal of Computational Chemistry* **2011,** *33* (5), 580-592.

9. Wang, R.; Bi, S.; Presser, V.; Feng, G., Systematic comparison of force fields for molecular dynamic simulation of Au(111)/Ionic liquid interfaces. *Fluid Phase Equilibria* **2018,** *463*, 106-113.

10. Wang, Y.; Tian, G., The Influence of Anion Structure on the Ionic Liquids/Au (100) Interface by Molecular Dynamics Simulations. *Langmuir* **2021,** *37* (48), 14059-14071.

11. Van Der Spoel, D.; Lindahl, E.; Hess, B.; Groenhof, G.; Mark, A. E.; Berendsen, H. J. C., GROMACS: Fast, flexible, and free. *Journal of Computational Chemistry* **2005,** *26* (16), 1701-1718.

12. Abraham, M. J.; Murtola, T.; Schulz, R.; Páll, S.; Smith, J. C.; Hess, B.; Lindahl, E., GROMACS: High performance molecular simulations through multi-level parallelism from laptops to supercomputers. *SoftwareX* **2015,** *1-2*, 19-25.

13. Duan, Y.; Wu, C.; Chowdhury, S.; Lee, M. C.; Xiong, G.; Zhang, W.; Yang, R.; Cieplak, P.; Luo, R.; Lee, T.; Caldwell, J.; Wang, J.; Kollman, P., A point‐charge force field for molecular mechanics simulations of proteins based on condensed‐phase quantum mechanical calculations. *Journal of Computational Chemistry* **2003,** *24* (16), 1999-2012.

14. Jorgensen, W. L.; Chandrasekhar, J.; Madura, J. D.; Impey, R. W.; Klein, M. L., Comparison of simple potential functions for simulating liquid water. *The Journal of Chemical Physics* **1983,** *79* (2), 926-935.

15. Martínez, L.; Andrade, R.; Birgin, E. G.; Martínez, J. M., PACKMOL: A package for building initial configurations for molecular dynamics simulations. *Journal of Computational Chemistry* **2009,** *30* (13), 2157-2164.

16. Hess, B.; Bekker, H.; Berendsen, H. J. C.; Fraaije, J. G. E. M., LINCS: A linear constraint solver for molecular simulations. *Journal of Computational Chemistry* **1997,** *18* (12), 1463-1472.

17. Xu, L. J.; Lei, Z. C.; Li, J.; Zong, C.; Yang, C. J.; Ren, B., Label-free surface-enhanced Raman spectroscopy detection of DNA with single-base sensitivity. *J Am Chem Soc* **2015,** *137* (15), 5149-54.

18. Guerrini, L.; Krpetic, Z.; van Lierop, D.; Alvarez-Puebla, R. A.; Graham, D., Direct surface-enhanced Raman scattering analysis of DNA duplexes. *Angew Chem Int Ed Engl* **2015,** *54* (4), 1144-8.

19. Abid Hasan, S. M.; He, Y.; Chang, T.-W.; Wang, J.; Gartia, M. R., Detecting DNA Methylation Using Surface-Enhanced Raman Spectroscopy. *The Journal of Physical Chemistry C* **2018,** *123* (1), 698-709.

20. Li, X.; Yang, T.; Li, C. S.; Song, Y.; Wang, D.; Jin, L.; Lou, H.; Li, W., Polymerase chain reaction - surface-enhanced Raman spectroscopy (PCR-SERS) method for gene methylation level detection in plasma. *Theranostics* **2020,** *10* (2), 898-909.
